# Re-Examination of Globally Flat Space-Time

**DOI:** 10.1371/journal.pone.0078114

**Published:** 2013-11-08

**Authors:** Michael R. Feldman

**Affiliations:** Private researcher, New York, New York, United States of America; University of Adelaide, Australia

## Abstract

In the following, we offer a novel approach to modeling the observed effects currently attributed to the theoretical concepts of “dark energy,” “dark matter,” and “dark flow.” Instead of assuming the existence of these theoretical concepts, we take an alternative route and choose to redefine what we consider to be inertial motion as well as what constitutes an inertial frame of reference in flat space-time. We adopt none of the features of our current cosmological models except for the requirement that special and general relativity be local approximations within our revised definition of inertial systems. Implicit in our ideas is the assumption that at “large enough” scales one can treat objects within these inertial systems as point-particles having an insignificant effect on the curvature of space-time. We then proceed under the assumption that time and space are fundamentally intertwined such that time- and spatial-translational invariance are not inherent symmetries of flat space-time (i.e., observable clock rates depend upon both relative velocity and spatial position within these inertial systems) and take the geodesics of this theory in the radial Rindler chart as the proper characterization of inertial motion. With this commitment, we are able to model solely with inertial motion the observed effects expected to be the result of “dark energy,” “dark matter,” and “dark flow.” In addition, we examine the potential observable implications of our theory in a gravitational system located within a confined region of an inertial reference frame, subsequently interpreting the Pioneer anomaly as support for our redefinition of inertial motion. As well, we extend our analysis into quantum mechanics by quantizing for a real scalar field and find a possible explanation for the asymmetry between matter and antimatter within the framework of these redefined inertial systems.

## Introduction

The purpose of this paper is to present the foundational groundwork for a new metric theory of *flat* space-time which takes into account the observed effects currently expected to be the result of ‘dark energy’ [1], ‘dark matter’ [2], and ‘dark flow’ [3] without resorting to these theoretical concepts that we have yet to observe in the laboratory. We emphasize above the fact that we are working in flat space-time as this paper is not concerned with reformulating gravity. Meaning, we assume gravity is the consequence of *local* curvature in space-time resulting from the energy-momentum content associated with an object as formulated by Einstein in his theory of general relativity. However, for our discussion, we operate under the assumption that at “large enough” scales we may treat massive objects in our proposed inertial reference frames as point-particles having an insignificant effect on the curvature of space-time for the purpose of examining the motion of said objects within the context of these larger scales. Consequently, we assume that *space-time* is essentially flat at these scales, and therefore, the energy density throughout our inertial systems is taken to be approximately zero. Thus, we assume that the deviation away from flat space-time inertial paths due to curvature in space-time is insignificant in our analysis. Furthermore, it is assumed that the observed motion of these large-scale objects about central points (e.g. stars orbiting the center of a galaxy, galaxies orbiting the center of a group/cluster, groups/clusters orbiting the center of a supercluster, etc.) is not due to the presence of gravitational sources at these centers but is instead a manifestation of the way in which objects move when no net external forces are acting upon them. In other words, the following work is concerned with reformulating our understanding of inertial motion. Furthermore, we focus on reformulating the global properties of an inertial reference frame while disregarding the potential local effects that objects moving within this global inertial system may have on the curvature of space-time. To begin with our reformulation, we explicitly state for the reader the assumptions of flat space-time as given by Einstein's special relativity [4]:

1. An object will travel in a straight line at a constant speed when no net external forces are acting upon this object (inertial motion adopted from Newton; see section titled “Definitions” in [5]).
2. An observer undergoing inertial motion has the freedom to describe events by “carrying rulers” in any three arbitrarily chosen spatial directions (perpendicular to one another) and calibrating clocks according to Einstein's prescription for synchronization (an inertial frame of reference). As well, inertial reference frames moving with uniform (constant velocity) rectilinear motion relative to one another are treated equally (i.e. there are no preferred inertial frames of reference in flat space-time).
3. The speed of light remains constant in all of these observer dependent inertial frames.

While operating under these assumptions in addition to those of general relativity [6], our cosmological models (e.g. 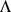
CDM [7]) then require a ‘Big-Bang’ event [8] [9] [10], ‘inflation’ [11], ‘dark energy’, ‘dark matter’, and ‘dark flow’ as explanations for observed phenomena on cosmological scales given our assumed understanding of inertial motion and inertial reference frames as stated above. In contrast, our claims in this paper are that in order to reproduce the observed behavior attributed to the theoretical concepts of ‘dark energy’, ‘dark matter’ and ‘dark flow’, it is not necessary to assume that these supplements must exist. Instead, it is possible to reproduce this behavior by simply incorporating it into a revised understanding of inertial motion and inertial reference frames in empty flat space-time, thereby no longer assuming the three pillars of theoretical physics as listed above and no longer requiring the occurrence of a ‘Big-Bang’, ‘inflation’, and expansion of space. While seemingly rash at first glance, we claim that in what we term as our “Theory of Inertial Centers”, as laid out in the following work, one can reproduce with inertial motion in our redefined inertial reference frames the following observed features:

1. Accelerated redshifts [12] and the Hubble relation [13].
2. Plateauing orbital velocity curves at large distances from a central point about which objects orbit [14].
3. Consistent velocity “flow” of objects toward a central point [3] [15].
4. An orientation associated with a particular frame of reference [16] (i.e. we do not take the cosmological principle to be a valid assumption as can be seen from experimental evidence such as [17]).

In our theory of flat space-time, inertial motion remains defined to be the motion of an object when it is subjected to no net external forces. In addition, an inertial reference frame is defined to be a system within which objects move along inertial trajectories when no net external forces are acting upon them. We then make the following assumptions and requirements in our theory:

1. Inertial motion is *not* characterized by an object moving in a straight line at a constant speed. Instead, inertial motion is characterized by geodesics about “inertial center points” in the radial Rindler chart as examined in the following discussion (the radial Rindler chart has been mentioned in other contexts such as [18] and [19]). Note that implicit in this assumption is the idea that time and space are fundamentally intertwined such that time-translational invariance and spatial-translational invariance are *not* inherent symmetries of flat space-time. Mathematically, this notion reduces to incorporating *both* time and spatial distance into the invariant interval associated with our metric. Meaning, the physically observable elapsed time as measured by a clock carried along a given curve, denoted as “proper time” 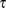, is *not* our affine parameter and thus is not invariant. Therefore, observable clock rates depend upon both spatial position in a particular inertial frame as well as in which inertial frame the observer is observing. Our affine parameter 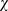 in the theory of inertial centers is then taken as a function of proper time in a particular inertial frame to be
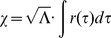
where 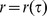 represents the physical distance to the inertial center about which the observer moves at a particular observable clock time 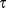 in the inertial system and 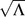 is taken to be the Hubble constant [13] [20]. In addition, these inertial center points define the centers of our inertial reference frames.
2. An observer does *not* have the freedom to describe an inertial reference frame in whichever way he/she chooses as in special relativity. We, as observers, are forced to adopt the *orientation* of the inertial reference frame that nature provides for us at the particular scale in which we are describing phenomena. As well, the inertial motion of an object must be thought of *relative* to the inertial center point about which said object orbits (throughout this paper, we will use the term “orbit” to refer to the inertial motion of an object about an inertial center point).
3. The speed of light is *not* constant throughout these inertial reference frames.
4. Locally within a confined region of each of these newly defined inertial reference frames, our theory reduces to and abides by the axioms of special relativity and general relativity.

Our analysis is organized in the following manner. First, we explore the limiting behavior of our equations of motion with the radial Rindler chart in flat space-time. Out of this, we come upon the ability to model the observed features as listed above. Second, we determine the limit in which our theory reduces to special relativity, while also proposing the form of our invariant interval in terms of both time and distance to an inertial center. We have stated the form of our affine parameter earlier in this introduction as a preface to the logic used in this proposition. Third, we examine the potential observable effects of this theory within our solar system and interpret the Pioneer anomaly [21] as support for our ideas. Fourth, we extend our analysis by quantizing our theory for a real massive scalar field. Within the context of this extension, we find a potential explanation for the asymmetry between matter and antimatter in our observable universe through the possibility of a parallel region to each inertial system embodied mathematically by the “other” radial Rindler wedge. We conclude by proposing future work including addressing the source of the cosmic microwave background [22] in this theory, attempting to explain other astrometric anomalies within our solar system besides Pioneer [23], and extending our quantum mechanical analysis to complex fields with spin.

## Discussion

### Geodesic paths

Adopting the signature 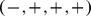 and employing abstract index notation throughout our analysis (see Chapter 2.4 of [24]), we work in the following metric:
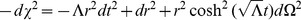
(1)where 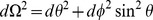; 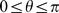, 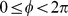, 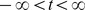, 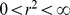, and 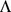 is a positive constant. In a subsequent section, we'll deduce that 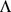 must be the square of the Hubble constant. 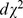 denotes the invariant interval associated with this metric where 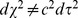 assuming 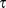 denotes proper time, defined as the physically observable elapsed time between two events as measured by a clock passing through both events carried along a particular curve, and 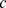 denotes the constant associated with the speed of light in special relativity. Therefore, in contrast with special relativity, our proper time interval is *not* assumed to be invariant, and the speed of light in flat space-time is *not* assumed to be constant. However, in subsequent sections, we shall show how special relativity can be treated as a local approximation to our theory of inertial centers. As in special and general relativity, massless particles travel along null geodesics. Thus, with this radial Rindler chart as the description of our inertial frame of reference and our redefinition of the invariant interval associated with the metric, we implicitly assume that time and space are fundamentally intertwined such that time-translational invariance and spatial-translational invariance are *not* inherent symmetries of flat space-time. In other words, one cannot progress coordinate time 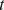 forward (i.e. replace 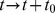 where 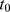 is a constant) without considering the effect of this action on space and vice versa. As well, this concept requires that we incorporate into the invariant interval associated with our metric *both* distance to inertial centers as well as proper time. Later in our analysis, we will express 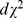 for this theory of inertial centers in terms of the proper time interval in a particular inertial frame.

For the affine connection terms, Ricci tensor elements, curvature scalar and square Riemann tensor, we refer to Appendix A in Appendix S1. From these calculations it is clear that this space-time geometry is indeed flat. Taking the Rindler transformation equations, 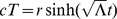, 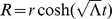, we find our metric equation becomes
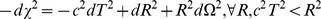
where 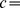 speed of light in the local Minkowski reference frame [25]. If one operates under the assumptions of special relativity, 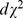 would in fact equal 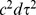, and then the metric in (1) can be used to model uniformly radially accelerated motion with respect to Minkowski space-time confined to either of the Rindler wedges: left wedge for 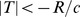 and right wedge for 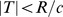 [26]. For the rest of our analysis, however, we no longer assume that special relativity is valid throughout globally flat space-time (again, 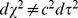) and instead examine the geodesic motion of point-particles in this radial Rindler coordinate system with time and radial distance from our inertial center point corresponding to the coordinate labels 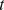 and 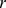, respectively. Additionally, as 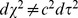, we do not assume that the reference frame itself is radially accelerating. Instead, we are re-examining inertial motion under the guidelines presented in our introduction keeping in mind that the form of our invariant interval is different from that of special and general relativity. And since our affine parameter is different from that of special and general relativity, the geodesics of our theory will also be different. Consequently, our employment of the radial Rindler chart in the following analysis is our way of establishing that this coordinate system is the “natural” one for describing an inertial system in the theory of inertial centers (i.e. coordinate time in the radial Rindler chart progresses at the same rate as the physical clock of a stationary observer in the inertial system). Thus, in the following work, *we abandon the idea that Minkowski coordinates can cover all of an inertial system in flat space-time*. Furthermore, we propose that *the radial Rindler chart should be our “natural” coordinate system for describing an inertial frame of reference in the theory of inertial centers*.

Referring to Appendix B in Appendix S1, we find for the equations of motion of a particle within a particular inertial system (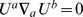, where our ‘proper velocity’ in component form is 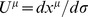):
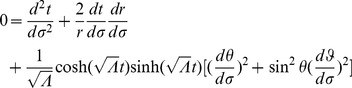
(2)

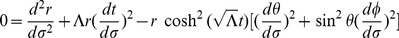
(3)

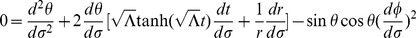
(4)

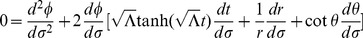
(5)And our norm for the ‘four-velocity’ is given by
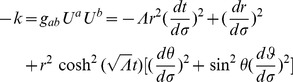
(6)where
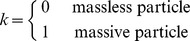
and 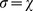 for massive particles. Notice that our ‘four-velocity’ 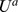 in this theory is dimensionless for spatial components and has units of [time]/[distance] for our time component since 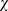 (and therefore 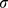) has units of [distance]. Multiplying each term in our radial equation of motion by 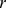 and plugging in (6),
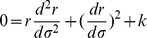
(7)But to remain at a constant radial distance away from our inertial center: 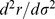, 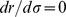. Therefore, only massless particles can have circular orbits.

Possible geodesic paths obey the relation 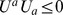 from (6), and solving for 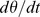 and 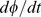, we find that
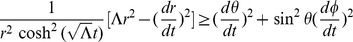
Examining our 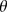 equation of motion (4), we see that a particle remains at a constant value of 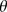 for non-zero angular velocity in 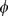 if and only if 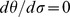 and 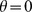, 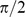, 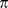. Consequently, the angular velocity of a particle traveling in the equatorial plane (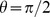) of this inertial reference frame is bound by the range:
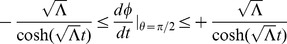
(8)Then, for a photon traveling in a circular orbit in the equatorial plane, we find
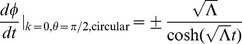
(9)Later, we'll see that a massless particle can have circular orbits only for 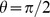 (orbits with 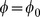 cannot be circular).

For massive particles nearly at rest with respect to the center of this inertial system (i.e. spatial ‘velocity’ terms are much smaller than our ‘velocity’ term in time so that these spatial terms can be taken as nearly zero), these four equations of motion (2), (3), (4), and (5) reduce to two:
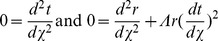
And solving for the radial acceleration, we find that
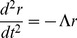
(10)In this limit, the inertial motion of our point-particle is described by a spatial acceleration in 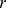 pulling inward toward the center of this particular reference frame scaled by the square of the time-scale constant. Thus, slowly moving objects at large radial distances experience a large radial acceleration pulling inward toward the center of the inertial system about which the objects orbit.

Then, let us examine the case where the motion of particles far from an inertial center (large 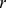) is dominated by angular velocities with approximately circular radial motion (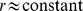). Our equations of motion reduce to
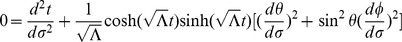


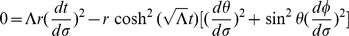


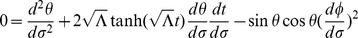


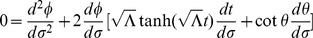
Plugging 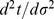 and 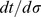 into our expressions for 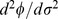 and 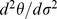:
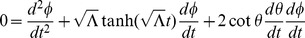


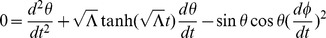
Now, if we assume 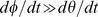 and integrate:
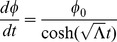
where 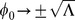 for light taking a circular orbit in the equatorial plane and thus 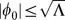 for 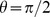. Plugging in for 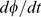, we have
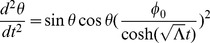
(11)where 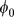 is a constant. In the large 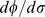 limit:
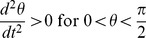
(12)

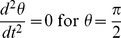
(13)

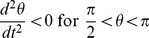
(14)As long as the particle is not located at either of the poles (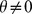, 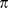), we see a sinusoidal spatial angular acceleration that decreases with 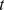 and moves the object toward 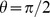. One can then picture spiral galaxy formation resulting from objects orbiting an inertial center with large angular velocity in 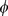.

If we refer back to our expression for 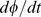, we find for the orbital velocity (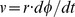) of a particle in this limit:
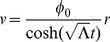
(15)And for 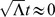, our particle's speed is linearly proportional to its radial distance away from the inertial center about which it orbits. In this limit at large 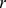, the relationship between orbital velocity and radial distance mimics the relationship between orbital velocity and radial distance found in our observed galaxy rotation curves [14] for comparably small values of 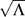 and therefore 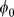. However, the analysis above will apply to the classical (in the sense that we are not taking into account quantum mechanics) inertial motion of an object in any particular inertial system (e.g. galaxies, groups, clusters, etc.). Later in our analysis, we'll provide an experimental scale for the time-scale constant 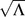 by analyzing the inherent redshift that occurs in these inertial frames (i.e. we'll take 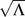 to be the Hubble constant). Since 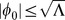, this value will also give us an upper limit for the slope of our orbital velocity curves at large 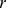. Thus, we claim that the linear relationship found in (15) models the experimental relationship found from our observed orbital velocity curves for objects far from the center of the galaxy within which they orbit. We base this claim off of the idea that the plateauing nature of our experimental curves would be interpreted in our model to be the result of the small scale of 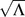 relative to galactic distance and orbital velocity scales.

### Conservation laws

Since this metric is just a coordinate transformation away from Minkowski, we expect to find ten linearly independent Killing vector fields as vector fields are geometric objects independent of our coordinate parametrization. One could obtain these using the radial Rindler transformation equations, but we find it helpful to explicitly derive them. We refer to Appendix C in Appendix S1 for more detail as well as a full list of all Killing vector fields given in the radial Rindler chart. Rewriting here for reference the three we will be using:
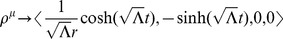
(16)

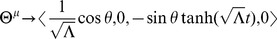
(17)

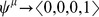
(18)Applying Noether's theorem (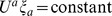),
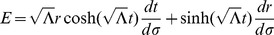
(19)

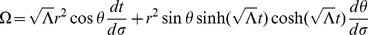
(20)

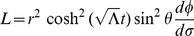
(21)Plugging into (6) and solving for 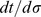, we find that
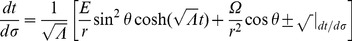
(22)where
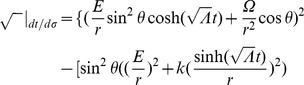


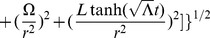
(23)requiring
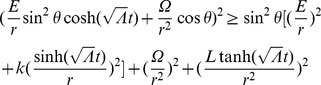
Notice, for massive particles moving radially in the equatorial plane (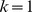, 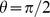, 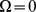, and 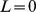), this constraint reduces to:
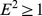
which is just our analogue of the statement in special relativity that the energy of an object must be greater than or equal to its rest mass [27] since in special relativity one would assume this constant 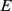 would equal the energy of the particle divided by its rest mass (i.e. in special relativity, 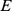 would be equal to 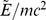 where 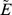 is the energy of the particle). Using (19), (20), (21), and (22), we find
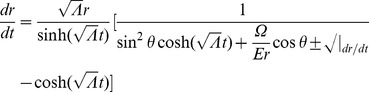
(24)

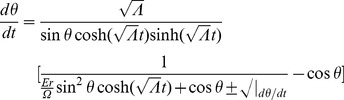
(25)

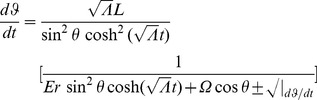
(26)where
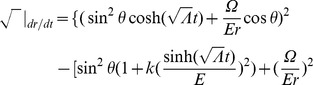


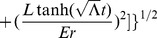
(27)

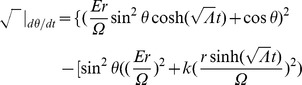


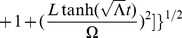
(28)

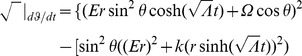


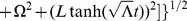
(29)For light traveling radially in the equatorial plane, 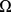, 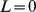 and (24) reduces to
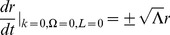
(30)giving us
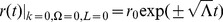
(31)where 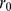 is a constant signifying the radial position of the photon at 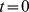. One could have arrived at this expression for the general case of light traveling radially even outside of the equatorial plane simply from (6) for null geodesics. Let us now pose the question of whether or not it is possible for light to travel from the 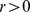 region of our inertial system to 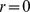 which we regard as our inertial center point. Integrating 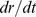 from 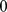 to 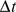,
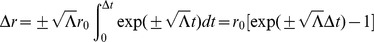
Solving for 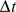,
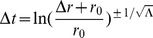
(32)For a photon traveling radially inward, the sign of the root is negative, and it reaches 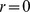 in
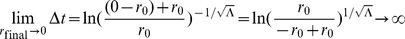
Consequently, not even light can reach 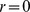 in a finite amount of time. But what about the inertial behavior of massive particles in these systems? At first glance, (24) and (25) appear to be divergent for 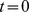. However, to evaluate all of these velocity expressions for 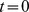, we return to symmetry equations (19) and (20):
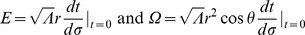
Therefore,
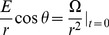
(33)Plugging into (23),
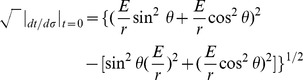


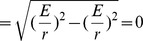
which one can plug back into (22) to find consistency with our expressions for 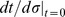 above. Yet we see from (27), (28), and (29) that
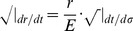
(34)

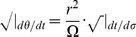
(35)

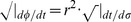
(36)which implies for 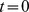 that all of these terms vanish. Then our spatial velocity terms for 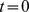 become
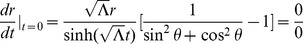


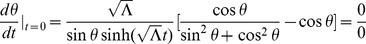


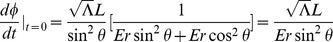
where we have used (33) in these limit expressions. So we see that our velocity terms are not necessarily divergent for 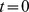. However, we'll address the issue of motion for small 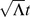 later when we relate Einstein's special relativity to our theory of inertial centers. One must also keep in mind that expressions (24), (25), and (26) represent a set of complex differential equations that we unfortunately will not be able to solve in this paper. The purpose of the following portion of this section is in fact to evaluate the large 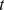 behavior of all spatial velocities where it is not explicitly apparent how to evaluate this limit if one were to work in Minkowski coordinates while keeping in mind the notion that he/she must relate back to the radial Rindler chart for inertial time as 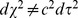 (we'll elaborate further on the term “inertial time” in our next section). It does appear easier to proceed in this manner of working in Minkowski and relating back to radial Rindler for solely radial motion as we shall do later in this section.

Yet, we return to our velocity expressions from Noether's theorem in order to examine the general expression for 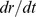 as 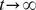. First, we determine the limiting value of 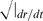:
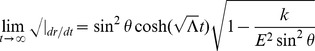
Then for massive particles (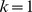) assuming 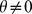 or 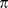,
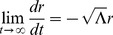
(37)which is just the equation for a massless particle traveling radially inward. When 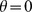 or 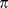, we must return to conservation equations (20) and (21). We find 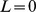 and
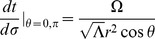
Plugging in (19) and solving when 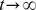, we again find (37). We now understand that eventually all massive particles move toward 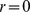. Yet as the object approaches the center, its speed decreases as well and will only stop moving inward when it reaches this inertial center point in an infinite amount of time. Thus, with this large 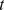 behavior, we apparently inherit the ability to model the observed anomalous effects of ‘dark flow’ [3]. In our next section, we will provide an interpretation for the physical significance of our coordinate time in our theory of inertial centers, relating 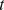 back to the rate at which physical clocks are observed to tick.

However, we progress onward and look at the large 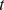 limits for both 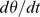 and 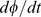. Beginning with the former, we find
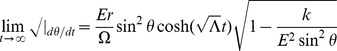
Plugging into 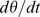 and examining for massive particles,
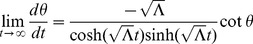
(38)And for 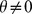 or 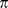,
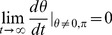
(39)Solving for 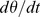 when 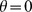 or 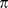 using (25) and 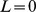,
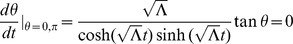
At the poles, particles have no angular velocity in 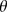 nor angular momentum in 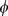 (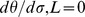). Lastly for our large 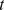 limits, we have 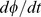:
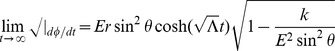
Plugging into 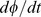,
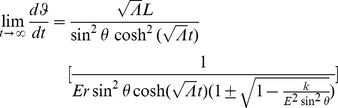
For massive particles and assuming 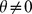 or 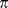,
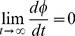
(40)We have a clearer picture of the inertial trajectories of massive particles over time in the context of our redefined inertial reference frames. As time progresses, massive objects will eventually move radially inward losing angular velocity in 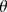 and angular momentum in 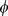, slowing down in radial velocity as they approach the center point about which they orbit.

Looking back at our expression for 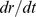, we ask ourselves the question: for what values of 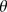 is 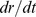 most positive? For positive 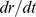, we have particles moving radially outward, and maximizing this expression with respect to 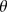 provides us with the easiest possible path to be ejected away from our inertial center. Examining particles with large radial ‘proper velocities’ relative to their own angular ‘proper velocities’ which from (19), (20), (21) implies 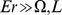 since 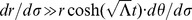 and 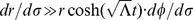 in this limit:
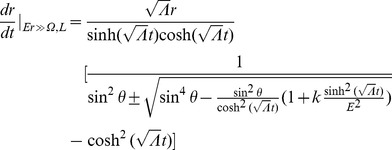
But the largest positive value of 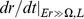 occurs if we minimize the denominator of the first term in brackets with respect to 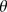. Clearly, this term needs to be re-evaluated when 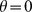 or 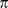. Returning to conservation equations (19) and (20), we solve for the radial motion of a particle through the poles by plugging into (6) (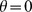 or 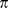 and 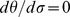):
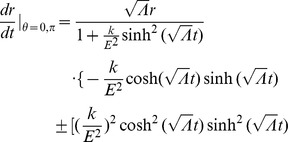


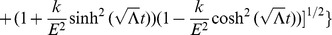
For large proper radial motion, we assume 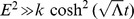 (as 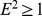 is our analogue of the rest mass condition from Einstein's special relativity). Then, our expression for radial motion through the poles reduces to
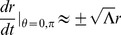
(41)We see that massive particles can travel at speeds near that of photons through the poles, and therefore it appears that the easiest way for particles to be ejected radially outward away from an inertial center would be through the poles of the inertial system. If we imagine a supernova occurring near the center point of an inertial system, we find that a simple potential scenario for the occurrence of relativistic jets [28] in this reference frame would be the expulsion of stellar remnants through the poles. Consequently, if we use this logic to provide an alternative for relativistic jet production, we must then require that each of our inertial frames have a particular orientation governed by the location of these poles and embodied mathematically by the spatial positions for which particular metric components vanish. In other words, when describing a particular inertial frame, these are the 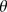 values for which 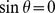 previously referred to as “coordinate singularities” (e.g. see Chapter 5.1 of [29]) but taken here as a physical attribute of the inertial system reflecting the idea that the radial Rindler chart is the “natural” coordinate system for an inertial reference frame in flat space-time. Thus we must ask ourselves the following question. How is this orientation established in the theory of inertial centers? As we shall mention later in our paper, this is an open question which we will have to address in future work.

Back to our circular orbit analysis, we solve for the radius at which light can have circular paths in a particular inertial system for possibly both 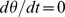, 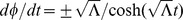 and 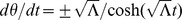, 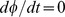. For the two, we obtain from (19)
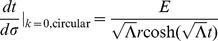
(42)In the former situation (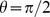, 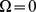), we substitute (42) and (21) into (6) and arrive at
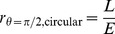
(43)Whereas for the latter case (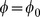, 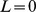), we plug (42) and (20) into (6) to find
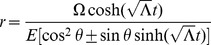
which is not constant. Consequently, in our inertial systems, light can travel in circular orbits only in the equatorial plane with angular velocity given by (9) at a radius given by (43). The type of lensing expected from a black hole or ‘dark matter’ [30] is evidently reproduced in a similar manner by light traveling with angular velocity about an inertial center point. Although in the analysis above, we studied circular orbits where light remains at a constant 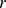, the logic applies similarly for the case where the photon has both radial and angular velocity components.

We come to the redshift factor for light traveling radially. Before we begin with this analysis, we must refer back to our procedure for determining the observed wavelength of a photon when operating under the assumptions of special and general relativity. In general relativity, the observed frequency 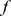 of a photon with momentum 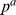 (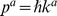) emitted/received by an observer traveling with proper velocity in component form given by 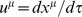 is (see Chapter 6.3 of [24])
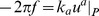
where 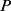 is the location in space-time at which the event in question occurs (i.e. emission/absorption). Dividing through by the Minkowski constant for the speed of light 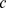, we have
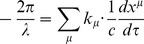
where 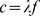 and 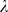 is the wavelength of the photon emitted/received by our observer. Since we require that our theory in flat space-time reduce to special relativity within a localized region of our respective inertial system (i.e. 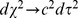 in this localized region), it appears necessary to assume that, in our theory of inertial centers, the wavelength of a photon with wave-vector 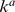 emitted/received by an observer with ‘proper velocity’ 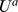 is given by
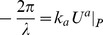
where we emphasize to the reader that in our theory the component form of the ‘four-velocity’ for our observer is affinely parametrized by 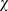 (i.e. 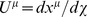), in direct contrast to special and general relativity for which the four-velocity of an observer would be affinely parametrized by proper time 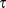. Proceeding with our radial treatment, the wave-vector for this photon is of the form,
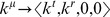
And the wavelength observed by a radially traveling individual is given by
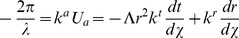
Using the Killing vector field in (16), we obtain the conservation law (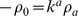):
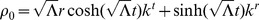
But for a photon, 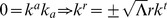. So,
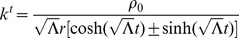
where the positive root corresponds to light traveling away from 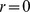 and negative to light traveling inward. Solving for the motion of the observer in this particular inertial reference frame,
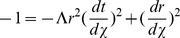
And for an observer nearly at rest with respect to the inertial center about which he/she orbits,
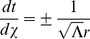
Taking time to move forward, we find that
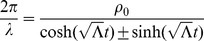
But from our earlier analysis, we found that a radially traveling photon abides by the equation, 
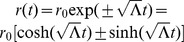
. Thus,
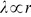
(44)Then for a light signal sent between two observers at rest in this inertial frame, the redshift factor 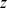 is given by the expression:
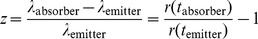
(45)Consequently, we see large shifts from emitters much closer to the center of the system (assuming the absorber position remains the same).

Suppose, within the framework of this theory, we examine light propagating at the scale of the inertial reference frame associated with our observable universe. Then analogous to the manner in which the expression for the Hubble parameter [13] is derived in the Friedmann-Lemaître-Robertson-Walker (FLRW) metric [31] [32] [33], we set the Hubble constant 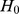 equal to
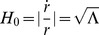
(46)where 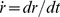. We notice that
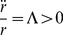
(47)producing a positive value for the acceleration of cosmological redshift and therefore replicating the observed effects assumed to be the result of ‘dark energy’ [12]. Thus, for any particular inertial reference frame, we should see a shift in wavelength similar to the Hubble constant for the radial motion of photons. We'll use this conclusion later when we take the Pioneer anomaly as support for our theory in the context of the inertial system associated with the Milky Way. However, we should be concerned with our expression for 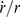,
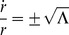
as this theory then requires that we also have blueshifted objects if the absorber is in fact closer than the emitter to the center of the inertial frame within which the light signal in question propagates (i.e. negative values for 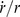 and 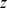). Nevertheless, if we apply our analysis to objects at the scale of the Local Group [34] [35] as in Table 1, we would require an alternative interpretation for the observed significant blueshifts. Whereas in current models, this blueshift would be interpreted as the Doppler effect and thus for example as Andromeda (Messier 31) moving with velocity toward the Milky Way [36], in our theory of inertial centers one could interpret a portion of this blueshift (we say portion as the motion of our observers within an inertial system also affects wavelength) as the possibility that Andromeda is farther away from the inertial center associated with the Local Group than we are. In support of these observations, we refer to Table 2 where there appears to be an orientation associated with our redshift values. For similar values of right ascension (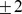 h), we see a steady change in wavelength shift from blue (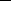) to red (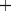) as one proceeds from large positive values of declination to large negative values of declination. In our theory, we would still need to consider differences in radial distance associated with these objects and not just spatial orientation. However, given that our distance modulus values are very much similar for most of these entries (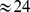 mag), it seems that this interpretation for an orientation to the Local Group should be taken into consideration. On the other hand, even if there does appear to be an orientation associated with the Local Group, we must question why we have not seen significant blueshifts at much larger scales. We will come back to these ideas later in our work.

**Table 1:** Redshift from objects within the Local Group.

Object	RA (J2000.0)	Dec (J2000.0)	Redshift	Distance Mod (mag)
Andromeda V	01h10m17.10s	+47d37m41.0s	−0.001344	24.52
Andromeda I	00h45m39.80s	+38d02m28.0s	−0.001228	24.46
Andromeda VI	23h51m46.30s	+24d34m57.0s	−0.001181	24.58
Andromeda III	00h35m33.78s	+36d29m51.9s	−0.001171	24.38
IC 0010	00h20m17.34s	+59d18m13.6s	−0.001161	24.57
Andromeda VII	23h26m31.74s	+50d40m32.6s	−0.001024	24.7
MESSIER 031	00h42m44.35s	+41d16m08.6s	−0.001001	24.46
Draco Dwarf	17h20m12.39s	+57d54m55.3s	−0.000974	19.61
Pisces I	01h03m55.00s	+21d53m06.0s	−0.000956	24.5
UMi Dwarf	15h09m08.49s	+67d13m21.4s	−0.000824	19.3
MESSIER 110	00h40m22.08s	+41d41m07.1s	−0.000804	24.5
IC 1613	01h04m47.79s	+02d07m04.0s	−0.000781	24.33
NGC 0185	00h38m57.97s	+48d20m14.6s	−0.000674	24.13
MESSIER 032	00h42m41.83s	+40d51m55.0s	−0.000667	24.42
NGC 0147	00h33m12.12s	+48d30m31.5s	−0.000644	24.3
Andromeda II	01h16m29.78s	+33d25m08.8s	−0.000627	24.03
Pegasus Dwarf	23h28m36.25s	+14d44m34.5s	−0.000612	26.34
MESSIER 033	01h33m50.89s	+30d39m36.8s	−0.000597	24.69
Aquarius dIrr	20h46m51.81s	−12d50m52.5s	−0.00047	26
WLM	00h01m58.16s	−15d27m39.3s	−0.000407	25.09
Cetus Dwarf Spheroidal	00h26m11.03s	−11d02m39.6s	−0.00029	24.51
SagDIG	19h29m59.58s	−17d40m51.3s	−0.000264	25.03
NGC 6822	19h44m57.74s	−14d48m12.4s	−0.00019	23.41
Leo A	09h59m26.46s	+30d44m47.0s	0.000067	
Fornax Dwarf Spheroidal	02h39m59.33s	−34d26m57.1s	0.000178	20.7
Phoenix Dwarf	01h51m06.34s	−44d26m40.9s	0.000187	23.08
Leo B	11h13m28.80s	+22d09m06.0s	0.000264	21.67
Sculptor Dwarf Elliptical	01h00m09.36s	−33d42m32.5s	0.000367	19.67
Sagittarius Dwarf Spheroidal	18h55m19.50s	−30d32m43.0s	0.000467	17.17
Small Magellanic Cloud	00h52m44.78s	−72d49m43.0s	0.000527	18.95
Tucana Dwarf	22h41m49.60s	−64d25m10.0s	0.000647	24.74
Sextans Dwarf Spheroidal	10h13m02.96s	−01d36m52.6s	0.000747	19.73
Carina Dwarf	06h41m36.69s	−50d57m58.3s	0.000764	20.02
Large Magellanic Cloud	05h23m34.53s	−69d45m22.1s	0.000927	18.46
Leo I	10h08m28.10s	+12d18m23.0s	0.000951	21.91

Data retrieved from NASA/IPAC Extragalactic Database (NED): http://ned.ipac.caltech.edu

**Table 2:** Redshift from galaxies within 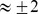 h of 0 h in RA within Local Group.

Object	RA (J2000.0)	Dec (J2000.0)	Redshift	Distance Mod (mag)
Andromeda V	01h10m17.10s	+47d37m41.0s	−0.001344	24.52
Andromeda I	00h45m39.80s	+38d02m28.0s	−0.001228	24.46
Andromeda VI	23h51m46.30s	+24d34m57.0s	−0.001181	24.58
Andromeda III	00h35m33.78s	+36d29m51.9s	−0.001171	24.38
IC 0010	00h20m17.34s	+59d18m13.6s	−0.001161	24.57
Andromeda VII	23h26m31.74s	+50d40m32.6s	−0.001024	24.7
MESSIER 031	00h42m44.35s	+41d16m08.6s	−0.001001	24.46
Pisces I	01h03m55.00s	+21d53m06.0s	−0.000956	24.5
MESSIER 110	00h40m22.08s	+41d41m07.1s	−0.000804	24.5
IC 1613	01h04m47.79s	+02d07m04.0s	−0.000781	24.33
NGC 0185	00h38m57.97s	+48d20m14.6s	−0.000674	24.13
MESSIER 032	00h42m41.83s	+40d51m55.0s	−0.000667	24.42
NGC 0147	00h33m12.12s	+48d30m31.5s	−0.000644	24.3
Andromeda II	01h16m29.78s	+33d25m08.8s	−0.000627	24.03
Pegasus Dwarf	23h28m36.25s	+14d44m34.5s	−0.000612	26.34
MESSIER 033	01h33m50.89s	+30d39m36.8s	−0.000597	24.69
WLM	00h01m58.16s	−15d27m39.3s	−0.000407	25.09
Cetus Dwarf Spheroidal	00h26m11.03s	−11d02m39.6s	−0.00029	24.51
Fornax Dwarf Spheroidal	02h39m59.33s	−34d26m57.1s	0.000178	20.7
Phoenix Dwarf	01h51m06.34s	−44d26m40.9s	0.000187	23.08
Sculptor Dwarf Elliptical	01h00m09.36s	−33d42m32.5s	0.000367	19.67
Small Magellanic Cloud	00h52m44.78s	−72d49m43.0s	0.000527	18.95

Data retrieved from NASA/IPAC Extragalactic Database (NED): http://ned.ipac.caltech.edu

**Figure 1:**
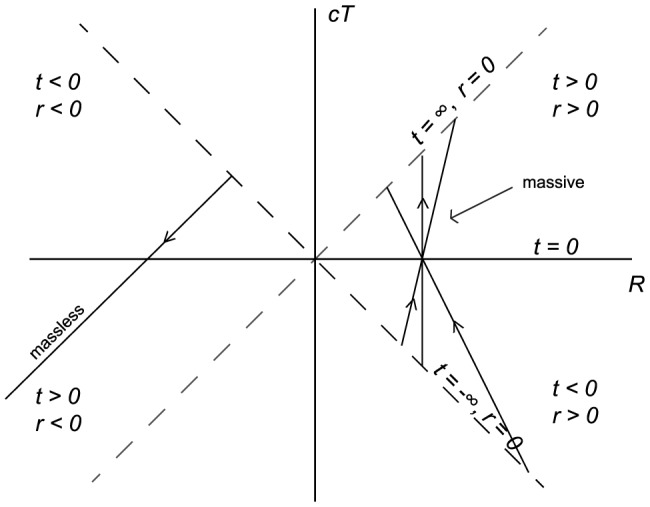
Geodesic paths in Minkowski coordinates.

Until now, we have assumed that our coordinate time can take values between 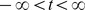 without explicitly examining the motion of particles in the 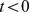 region. Reducing our analysis to solely radial motion away from the poles, we analyze geodesic paths in Minkowski coordinates (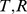) first for simplicity. However, we must be very clear that under our assumptions 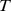 does not represent inertial time as previously stated and in our theory of inertial centers corresponds to an “unnatural” time coordinate for flat space-time combining both physically observable clock time and spatial distance as 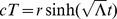. Then our equations of motion reduce to
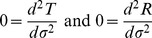
leading to the straight lines that we expect in Minkowski coordinates:
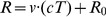
(48)where 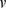 is a constant bounded by 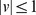. We leave the physical interpretation of the Minkowski constant 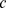 in this theory of inertial centers for the next section. However, using our transformation equations, we find in radial Rindler coordinates
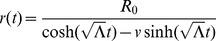
(49)One immediately notices that for massive particles (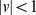), both limiting cases of 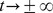 result in the particle heading inward toward the 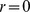 center point of the inertial system. This produces a scenario for inertial motion of massive objects beginning at a center point in the far past, coming to a maximum radial distance away at a later time, and then heading back inward to eventually return to the same center point. In other words, classically, all particles must also originate from the 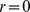 center point of the particular inertial frame in question (see Figure 1).

### Reduction to special relativity

Taking the differential of both Rindler transformation equations:
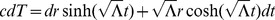


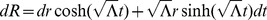
where
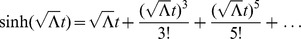


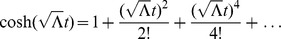
Plugging in these expressions above, we find that
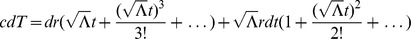


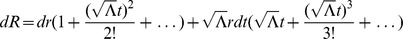
If we localize our view of space-time such that all differential terms
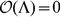
(50)we will then have
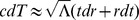


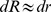
Further, we require for this local patch of space-time that
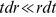
(51)and our transformation equations reduce to
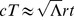
(52)

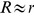
(53)with differential expressions
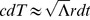
(54)

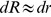
(55)


For the observer remaining a radial distance 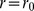 away from the center of his/her reference frame, the radial Rindler chart will be accurately approximated by Minkowski coordinates under conditions (50) and (51) as 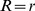 and 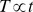 in this small 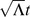 limit. If one takes 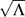 to be a fundamental property of each inertial system in question, it must be that the measured Minkowski value for the speed of light constant 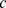 is a byproduct of the reference frame we wish to locally approximate. In other words, in the Minkowski approximation for the radial Rindler chart, an observer, located a radial distance 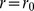 away from the inertial center point about which he/she orbits at 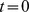, will find:
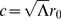
(56)If we treat 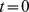 as the point at which we determine the initial conditions for the particle that we are observing (i.e. boundary conditions for position and velocity), then our object will appear to move along straight line geodesics for small values of 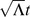, but as we continue to observe for longer periods of time, the properties of the radial Rindler chart which we are approximating become more and more relevant.

**Figure 2:**
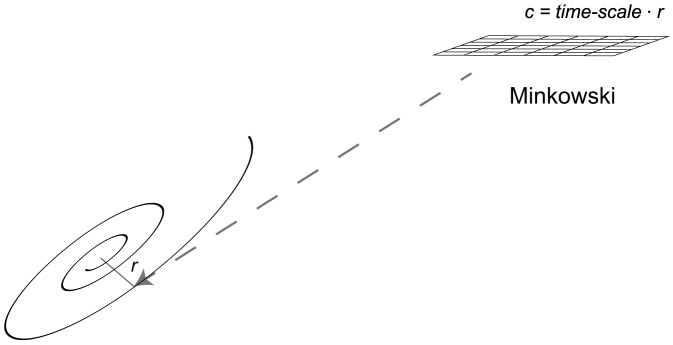
Local approximation of the inertial frame of reference.

In order for us to relate our theory of inertial centers to special relativity, we must require that coordinate time in the radial Rindler chart progress at the same rate as the proper time of an observer stationary relative to the center of the inertial system within which we are analyzing events (i.e. inertial time). In other words, 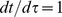 for stationary observers located at any particular radial distance 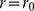 away from an inertial center. However, keep in mind that stationary observers do not follow along geodesic paths from equation (7). Then, for observers which we can consider as nearly stationary relative to the center of a particular inertial system (i.e. 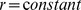), we have 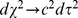 where 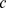 is given by (56), effectively ensuring that our coordinate time 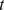 progresses at the same rate as the proper time 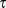 of a stationary observer. Consequently, we find under (50) and (51) in addition to our stationary observer assumption that our line element can be treated approximately as
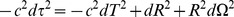
And therefore in this “stationary” limit (relative to the inertial center), when not operating about the poles, we come upon time- and spatial-translational invariance within our local region where the origin of our coordinate system is located at the inertial center of this reference frame. Because it appears that we have now recovered time- and spatial-translational invariance in this limit, we can naively assume that we have the ability to translate our coordinate system in any way we prefer (e.g. moving the center of our reference frame away from the inertial center). In other words, we can approximate when our motion is not near the poles of our global inertial system with the metric:
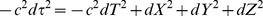
where 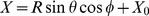, 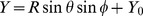, and 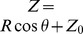 for 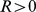 and 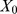, 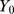, and 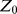 are constants (see Figure 2). Thus, this local stationary approximation reduces our theory to special relativity (see Chapter 4.2 of [24]). Additionally, we see from these transformation equations that the Minkowski chart is not able to cover all of space-time in our theory of inertial centers (i.e. 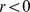 values are neglected by the Minkowski chart). We will come back to this idea later in our work. Physically, our localization conditions require that the time-scale constant 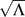 for our inertial systems be small enough such that we as observers here on Earth would observe only the stationary limit in our “everyday lives”. Of course, this statement also assumes that we are nearly stationary relative to the inertial center about which we orbit taken in our next section to be the center of the Milky Way. Yet, given our redshift analysis, it appears that the Hubble constant provides the necessary scale [20] for this requirement from (46).

Furthermore, it is clear that in order to express 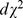 in terms of the proper time of the observer whose motion we wish to analyze in the relevant inertial frame (i.e. the particular system in which the observer can be treated as a point-particle orbiting an inertial center point) and still have our invariant interval reduce to 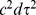 in our stationary limit where the observer's distance to the inertial center point about which he/she orbits is very nearly constant, we must have
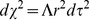
(57)where 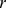 represents the physical distance to the center point of the inertial system in question. In addition, according to our theory of inertial centers, the value that we use in special and general relativity for the constant 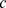 in our massive geodesic equations relies on the particular inertial reference frame in which we can regard the object whose local behavior we wish to examine as a point-particle orbiting an inertial center point (in special and general relativity, 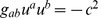 where 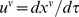). Therefore, the local Minkowski constant that we measure for the speed of light is dependent upon our position in our most local inertial reference frame (i.e. the frame in which we can be treated as a point-particle orbiting an inertial center). As well, for two different stationary observers orbiting about the same inertial center point, the clock of the observer located *closer* to their shared inertial center will appear to *run faster* when examined from the perspective of the more distant observer. Meaning, not only do observable clock rates differ due to the relative velocity of individuals as in special relativity, but they also differ due to the difference in distance of each individual from the inertial center about which each orbits. Thus, initially synchronized clocks that are stationary relative to the shared inertial center about which both orbit *do not remain synchronized* if they are located at different distances from this inertial center.

### Application to a local gravitational system

Before we present the approximations of this section, it seems necessary to provide remarks as to how gravitation fits into the theory of inertial centers. The formulation of our theory of inertial centers detailed in previous sections deals with the structure of flat space-time ignoring possible issues with curvature. So what we are really asking is the following. How does an object move in flat space-time when absolutely no external forces, fields, etc. are present to affect said object? Nevertheless, we still assume within our model that all objects cause curvature in space-time due to their intrinsic energy-momentum content, but this curvature we take to be a local effect within the far larger inertial system that we are attempting to redefine in this work. However, as presented in the previous section “Reduction to special relativity”, we claim that locally the structure of flat space-time within our redefined inertial reference frames reduces to the flat space-time of Einstein's theory of special relativity as long as the observer remains at very nearly the same distance away from the inertial center about which he/she orbits. As discussed above, we term this the “local stationary approximation”, and in this approximation the observer sees space-time locally within a region located at the same radial distance as the observer away from the inertial center about which he/she orbits as approximately special relativistic, where the speed of light in this confined region of space-time is given by (56) and our affine parameter reduces to 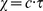. If one then considers the influence of an object on the structure of space-time in this local region where special relativity approximately holds, we assume in the theory of inertial centers that this object will bend space-time locally according to Einstein's general relativity. Meaning, gravity remains a consequence of *local* curvature in space-time in the theory of inertial centers. However, when we take a perspective far away from our massive object so that the curvature this object induces in space-time looks approximately insignificant for the purpose of examining motion at these larger scales, we claim that we can treat the object very nearly as a particle in a flat space-time inertial reference frame as formulated above, where the inertial motion of the object is dictated by the geodesics of our model. But again, if we focus our attention on the local region around the massive object while disregarding the existence of the larger inertial system, we will still observe the effects resulting from the curvature the object induces in space-time and thus the gravitational effects it has on other objects around it (i.e. general relativity holds locally).

In the following, we give an approximate method under our stationary localization conditions as described in the previous section for determining a potential implication of our theory of inertial centers with regard to the observables of a local gravitational system. We take the view that the Schwarzschild metric [37] applies in the small 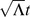 limit within confined regions of our inertial reference frame for observers nearly stationary with respect to the inertial center about which they orbit as one would expect from the well-established accuracy of general relativity [38]. Below, our “mixing” of the Schwarzschild metric with the radial Rindler chart is an approximate way of expressing the fact that locally in the inertial reference frame of our theory the observer can treat the speed of light as nearly constant if one were to remove the massive object and work in flat space-time (i.e. set 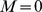 in the Schwarschild metric) as well as the idea that general relativity holds locally. But the observer must always keep in mind that the inertial frame of Einstein's special relativity is actually *not* an inertial frame of the theory of inertial centers, and thus this local system is located in the more globally relevant inertial system where the speed of light is not constant. Then if we no longer take 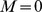 (i.e. return the massive object to the local system), we should still expect gravitation as formulated by Einstein when we examine locally and disregard the larger inertial system from our model. In other words, the Schwarzschild metric still applies locally in the theory of inertial centers when examining motion about an uncharged non-rotating spherically symmetric massive object. However, if we move our observer farther and farther away from the gravitational source, the local limit will no longer apply since we have to take into consideration the structure and properties of the larger inertial system as well as the fact that in our theory objects move inertially along geodesics different from those of Einstein's theories of special and general relativity, even though locally these different geodesics appear to be very similar (i.e. 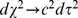 in the local stationary limit).

Additionally, we need some approximate way to take into account the fact that the speed of light is not constant throughout the inertial system while still keeping in mind that locally the observer may experience gravitational effects from a massive object nearby. We admit that the methods in this section are rough at best, but it is our hope that in future work we will be able to model far more accurately this transition from the local approximation of general relativity to the more global application of the theory of inertial centers.

Our metric equation takes the form of the Schwarzschild solution:
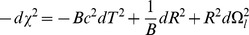
where
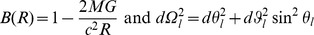
(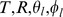) describe our local gravitational system and (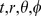) refer to the global inertial reference frame within which the local system is located. In other words, we assume that the observer takes the coordinate transformations away from the inertial center to cover local space-time in the same manner as outlined in our previous section. Meaning, ignoring the existence of the massive object
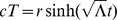


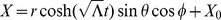


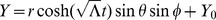


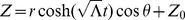
where 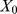, 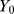, and 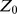 are constants, 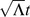 is taken to be small, and we only examine the 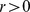 region of the inertial system. Thus, 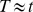 where we employ equation (56) for the local stationary limit. Then taking into account the existence of this massive object in the local region with 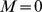 flat space-time Minkowski coordinates given by (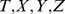) in the local stationary limit, we employ the Schwarzschild metric noting that our affine parameter is approximately given by 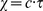. As well, 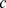 refers to the speed of light in the local system at the point in the global inertial frame where the observer and photon meet, and 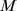 is the mass of the object. Then we will proceed through a standard treatment of the gravitational redshift for the Schwarzschild metric (see Chapter 6.3 of [24]). However, we keep the Minkowski constant 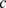 in all of our expressions as we intend to investigate the implications of the variable nature of the speed of light in flat space-time from our theory of inertial centers. For an observer and photon both traveling radially in this local system (
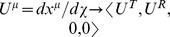
, 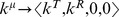), we have
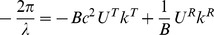
where 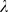 is the wavelength measured by our observer. Applying conservation laws for 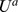 and 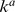 using the time-translationally invariant Killing vector field for the Schwarzschild metric, 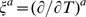:
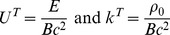
And taking into account the motion of the observer and photon (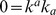 and 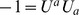)
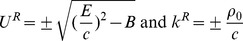
Plugging into our expression for the observed wavelength of the photon,
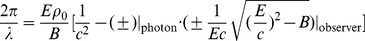
(58)where 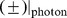 and 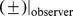 refer to the photon/observer traveling radially outward/inward (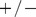) in the local system (in 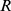). If we assume the observer to be nearly at rest in the local frame (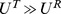), then 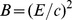 and expression (58) reduces to
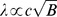
where in the following we approximate in the small 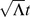 limit with our equation for the local speed of light in the inertial reference frame (56). We employ this “trick” as the Schwarzschild metric is just an approximation in our model valid under confined regions of the particular inertial system within which the gravitational source is located. However, one should be able to experimentally detect with an apparatus of the necessary sensitivity that these photons progress along the geodesics of our theory of inertial centers (and not straight lines) bent locally due to the curvature in space-time caused by our massive object 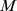. Therefore, we find a slight modification to the Schwarzschild redshift factor:
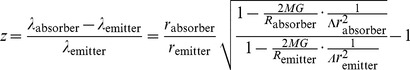
(59)where 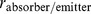 refers to the radial position of the absorber/emitter in the inertial reference frame (i.e. relative to the inertial center) and 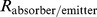 to the radial position relative to the center of our massive object 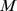 in the local gravitational system. Consequently, we should see a modified redshift factor consisting of the Schwarzschild expression (Chapter 6.3 of [24]) scaled by the solution found in our flat space-time vacuum analysis.

Let us then apply this analysis to the case of a space probe traveling out of our solar system where the 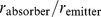 factor should have a larger impact on our observations. In our crude example, we treat both the probe and the absorber as essentially stationary. Referring to expression (59) for observers at rest, the absorber wavelength in terms of the emitter is
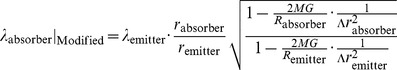
where our 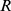 values in this example refer to local radial distances away from the center of the Sun and 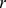 to distances away from the center of the Milky Way. For the ratio between the Schwarzschild wavelength and the modified value above assuming the term under the square root remains approximately the same for small changes in 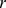 relative to changes in 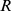, we have
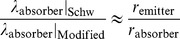
where
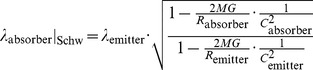
Since Pioneer 10 was on course to travel away from the center of the Milky Way in the general direction of Aldebaran [4], we can approximate the path of our photon as nearly a radial one in our galactic inertial reference frame. Therefore, if we naively ignore the two-way nature of the Doppler residuals, 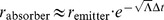 where 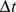 is the time it takes the massless particle to travel from the emitter to the absorber, assuming time measured by the emitter progresses at nearly the same rate as that measured by the absorber in this short distance calculation (i.e. 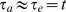). Notice, these photons travelled inward for Pioneer 10, so the root is negative. Plugging into our expression above, the fractional difference in wavelength predicted here on Earth is approximately
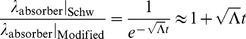
to first order where we assume that our modified expression coincides with our experimental values. Then the observed “time acceleration” reported in [39] and [40] provides an estimate for the time-scale of our galaxy of 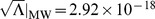 s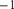. The consistency of this value with that of the Hubble constant [20] lends support to the argument that the time-scale 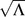 is universal for all inertial reference frames as we had implicitly assumed from our proposed form of the affine parameter presented in our introduction. However, further experiment is necessary in order to verify this claim.

Clearly, the two-way nature of the Doppler residuals of the Pioneer experiments as well as the difference in clock rates for varying positions within an inertial system in our theory will complicate our analysis further. However, the purpose of this section is to illuminate to the reader the idea that we may have evidence from experiments within our own solar system that support the relevance of this theory of inertial centers and suggest that possibly all inertial reference frames as defined within this theory abide by the same fundamental time-scale constant 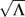. Nevertheless, others have argued as in [41] that the Pioneer anomaly is a consequence of the mechanics of the spacecrafts themselves instead of evidence of “new physics”. Therefore, to gain more support for the theory of inertial centers, we must address in future work not only the two-way nature of the Doppler residuals as both Pioneer 10 *and* Pioneer 11 appear to report blueshifted wavelengths even when they traveled in opposite directions with respect to the galactic inertial frame of reference but also the possibility that our theory can succinctly explain the other astrometric Solar System anomalies outlined in [23] and [40].

### Quantization of a real scalar field

We begin our extension into quantum field theory from the covariant form of the Klein-Gordon equation [42]:
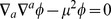
where 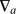 is the derivative operator compatible with the metric 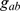 (i.e. 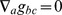), 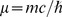, 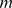 is the mass associated with our field, and 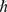 is the reduced Planck constant. First, we explore how one can intuitively arrive at this equation of motion given our classical assumptions. In special relativity, we have
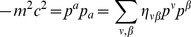
where 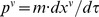 and 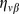 refers to the Minkowski metric components. Making the substitution 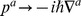, we come upon the Klein-Gordon equation above for a scalar field.

However, in our theory of inertial centers, the equation of motion in terms of ‘momentum’ is given by
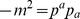
where now 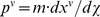 and so we have a major difference in our ‘momentum’ terms. In contrast with our experience in relativity, the ‘four-velocity’ for massive particles in our theory is parametrized by 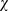 and *not* by proper time 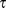. Unfortunately, there does not appear to be a natural operator substitution for 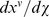. Yet, if we use expression (57), we have a potential extension of the Klein-Gordon equation when analyzing motion *at a particular scale*. It appears that one should substitute 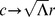 to find
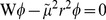
(60)where 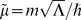 and 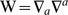 is the Laplace-Beltrami operator. Notice that in our equation of motion we have explicit reference to the particular inertial reference frame in which we are analyzing the behavior of the field as opposed to the Klein-Gordon equation which has no explicit reference to any inertial system. This seems to be consistent with the idea that the proper time is *not* the invariant quantity associated with our theory of inertial centers, and therefore our choice of proper time reflects the choice of *scale* in which we must work to analyze the progression of our field within this inertial system. One can also apply this substitution in an analogous manner to other equations of motion/Lagrangians, yet in the following we will only address the simple case of a free real massive scalar field.

Then, as outlined in Chapter 4.2 of [43] and briefly reviewed in Appendix D in Appendix S1, we must “slice” our manifold 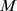 into space-like hypersurfaces each indexed by 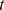 (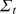). For our radial Rindler chart, the future-directed unit normal to each 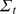 is given by
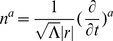
(61)where the absolute value is necessary to keep 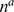 future-directed for all values of 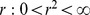, allowing for positive and negative values. We will interpret the physical significance of this relaxation on the domain restrictions for our radial coordinate later in our analysis. We see that our hypersurface can be decomposed into the union of two surfaces for each of the Rindler wedges (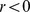 and 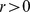), and thus the inner product of our Klein-Gordon extension is given by
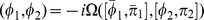


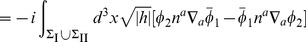
(62)where the bar symbol indicates complex conjugation (i.e. 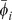 is the complex conjugate of 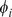), 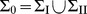 is the union of these two radial Rindler wedge space-like hypersurfaces, 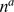 is the unit normal to our space-like hypersurface 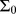, 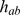 is the induced Riemannian metric on 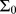 (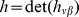; 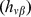 denotes the matrix associated with these Riemannian metric components), and 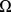 refers to the symplectic structure for our extension of the Klein-Gordon equation.

We should be rather concerned considering the discontinuous nature of the time-orientation of 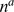 (the absolute value is not a smooth function) as well as the undefined behavior of our unit normal for 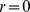, the location of our inertial center. However, given the solutions we find below, it seems to be an important question whether or not we are forced to treat each Rindler wedge separately as its own globally hyperbolic space-time or the combination of these wedges as the entire space-time over which we must analyze solutions to our extension of the Klein-Gordon equation. The difference between these two formulations will be that in the former we must define separate creation and annihilation operators for each wedge as in the analysis of [44]. Whereas in the latter, we have one set of creation and annihilation operators for all values of 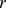 over the range: 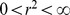, where 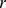 can take both positive and negative values. It also seems likely that a greater understanding of our inertial centers and their physical significance (i.e. how are these inertial centers established?) will provide far more insight into the proper way to treat this situation. In this paper, however, we assume the latter approach requiring that we use all values of 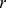 (positive and negative) to cover our inertial reference frame and naively ignore the issues with 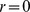 mentioned above. This approach seems to be far more consistent with the idea implicit in our theory of inertial centers that the radial Rindler chart covers the entire flat space-time manifold for the inertial system in question, except of course for the location of each of our inertial centers (i.e. 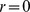). We find that our inner product is given by
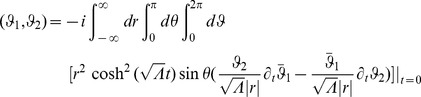


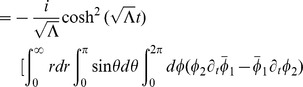


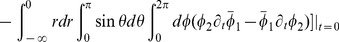
(63)Our remaining task reduces to solving for solutions (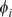) to our extension of the Klein-Gordon equation (60). From Appendix F in Appendix S1 which utilizes [45], [46], [47], [48], and [49], we find
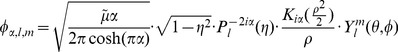
(64)where 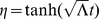 and 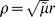. 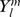 is the spherical harmonic of degree 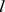 and order 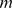. We maintain convention and use 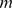 to denote the order of 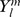. However, this 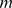 is a quantum number very different from the mass of our scalar field. The mass term is contained solely in our expression for 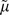. 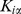 is the Macdonald function (modified Bessel function) of imaginary order 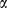. 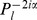 is the Legendre function of degree 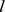 and imaginary order 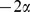.

**Figure 3:**
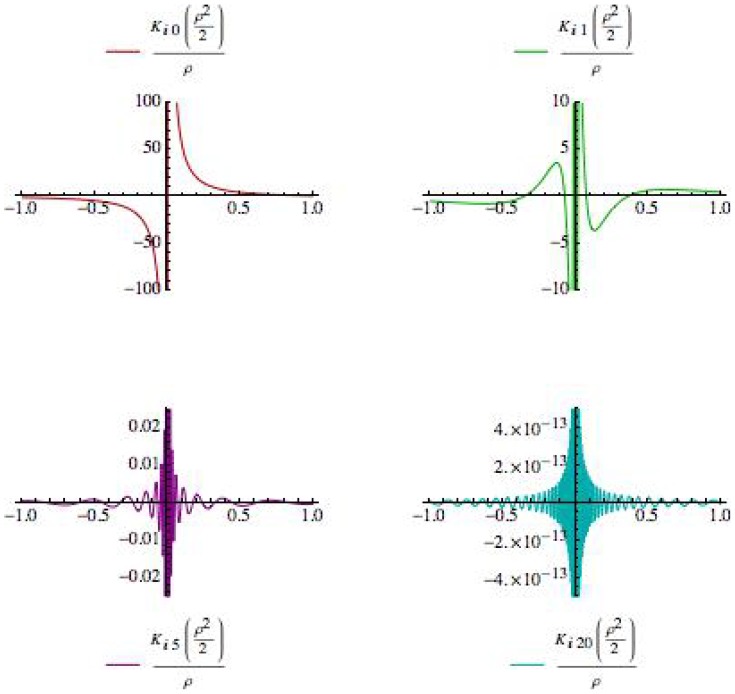
Plots of 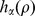 for 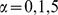, and 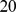.

Notice, we allow 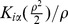 to have domain: 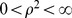 where 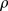 can take both positive and negative values. Physically, this interpretation requires the existence of the field in *both* the negative and positive 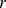 regions of the inertial system which brings us back to the discussion earlier in this section of our concern with 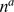. From [50], the limiting behavior of 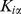 expressed as
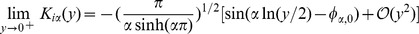
where 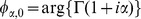 and 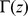 is the gamma function along with
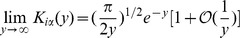
shows that 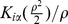 oscillates for small 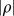 when 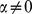 and exponentially decays for large 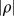. In addition, from Figure 3, we see that our radial ‘wave function’ spreads out away from 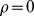 for larger ‘momentum’ values of 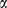, allowing for oscillatory behavior at larger values of 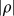 and thus an increased likelihood of observing quanta farther away from the inertial center of the reference frame in question.

Our Heisenberg field operator can be expanded in the following manner (see Chapters 3.1 and 3.2 of [43]):
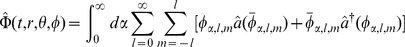


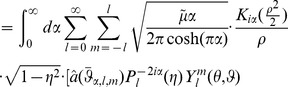


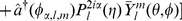
(65)where the annihilation and creation operators in terms of our inner product are
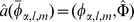


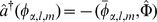
and 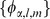 comprise an orthonormal basis of the “positive frequency” solutions to the extended version of the Klein-Gordon equation for the theory of inertial centers. For a real scalar field, these annihilation and creation operators satisfy the commutation relations (bosonic statistics):
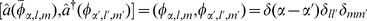



A very important point for the reader to take away from our analysis in this section is that our field operator as defined in (65) exists in *both* the 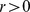 and 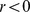 portions of space-time. In other words, we take space-time to be comprised of both the 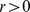 and 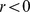 regions of the inertial system, and thus the Minkowski chart is *not* able to cover all of space-time in our theory. It then appears that a potential explanation for the matter/antimatter asymmetry in our observable universe within the framework of our theory of inertial centers would be that there exists a parallel region of each inertial system embodied mathematically above by the existence of our field operator in the hypothetical 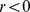 region of space-time. Logically, if we exist in our region of space-time with an imbalance toward matter, one would then assume that in this parallel region there exists an imbalance in favor of antimatter as the total charge of the field throughout *all* of space-time should be conserved. We are, of course, operating under the assumption that the solutions to our equation of motion extend in a similar manner as in special relativity when one allows for complex fields of non-zero spin (e.g. solutions to a Dirac equation [51] extension are also solutions to our Klein-Gordon extension) since we should not worry about antiparticles with a real scalar field. Therefore, we must extend our work on the theory of inertial centers to incorporate spin in order to see the full significance of this possible explanation for the matter/antimatter asymmetry in our observable universe.

To conclude our discussion, we assume throughout the rest of this section that 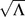 is a universal constant for all inertial systems, taken to be the Hubble constant as proposed in our introduction, and imagine that there exists an observer very near to an inertial center point such that his/her motion in this particular reference frame is approximately stationary (i.e. spatial ‘four-velocities’ are very much outweighed by ‘velocity’ in time, 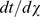). Then from our classical analysis of geodesic paths, our observer experiences a radial acceleration according to (10) of
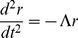
where 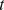 coincides with the proper time 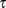 for our nearly stationary observer in this system. However, say we wish to understand our observer's motion *not* in terms of his/her proper time in this particular inertial frame but instead in terms of his/her proper time in an external inertial frame of reference where these two different systems do not share a common inertial center point. We know that our invariant interval is given by
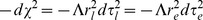
where the 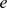 (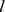) subscript refers to quantities in the external (local) inertial reference frame. Assuming our observer is nearly stationary in *both* inertial systems (i.e. coordinate times for each system coincide with proper times within each reference frame respectively), his/her clock in the local frame progresses by
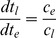
(66)Thus,
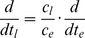
where the 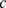
's refer to the Minkowski constants for each particular reference frame (56). Plugging in above,
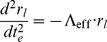
(67)where 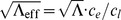.

According to Newtonian mechanics which is a good approximation here since we assume our observer is nearly stationary in the local inertial system, one would attribute this radial acceleration to a ‘force’ (even though we know that there really is no force here), and associated with this ‘force’ is a potential (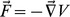; see Chapters 1 and 2 of [52]). So for the acceleration above, one would assume while working in Newtonian mechanics that there exists a potential causing this movement of the form:
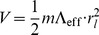
(68)Then our Hamiltonian (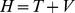; see Chapter 8 of [52]) for this system is given by
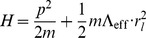
(69)where 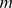 is the mass of our observer and 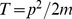 is the kinetic energy associated with his/her motion as observed in the external frame. If our observer is on the order of 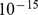 m [53] away from his/her local inertial center and 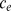 is found in the external frame to be 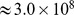 m/s [54], we find 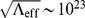 s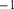. We remark for the reader less acquainted with nuclear theory that the Hamiltonian above is referred to as the isotropic harmonic oscillator and was used as a starting point for nuclear shell models due to its ability to reproduce the “magic numbers” associated with stable configurations of nucleons within the nucleus (see Chapter 4 of [55] and Chapter 3.7 of [56]). In addition, the energy scale associated with the Hamiltonian above (i.e. 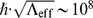 eV) is of a similar order as the scale inputted into these isotropic harmonic oscillator models for the magnitude of the nuclear ‘force’ [57]. Thus, our ability to replicate the same features as those of the simplest nuclear shell model compels us to ask the following question with regard to the theory of inertial centers: *Is there an inertial center point at the center of the nucleus of every atom?*

## Limitations of the study, open questions, and future work

There is a plethora of data for us to critically investigate the validity of this theory of inertial centers. Nevertheless, we have chosen to leave these detailed investigations for future work as the purpose of this paper is to lay out the theoretical foundations to illicit these types of rigorous comparisons with experiment for all aspects of our model. As we have mentioned briefly at certain points within our discussion, there are many open questions that must be addressed. The most pressing of these appears to be how to explain the cosmic microwave background (CMB) within our theory of inertial centers. One may be tempted to immediately point to the Fulling-Davies-Unruh effect [58] as the source of this cosmic radiation since the Unruh effect predicts that an “accelerating” observer in Minkowski vacuum, who can be described by orbits of constant spatial coordinate in the classic Rindler chart, detects black-body radiation that appears to be nearly homogeneous and isotropic with predicted anisotropies due to the orientation of this observer throughout his/her “accelerated” path [59]. However, we must keep in mind that the scale associated with the temperature of Unruh radiation [58] [44]

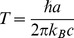
requires 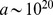 m/s^2^ to produce a temperature on the order of the CMB, 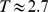 K [60], where 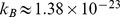 J K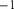 is Boltzmann's constant, 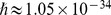 J s [54], and 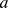 is the proper acceleration of the observer. If we approximate the original analysis of [58] by working in 1+1 space-time (i.e. 1 time and 1 spatial dimension), the acceleration would be proportional to the inverse of 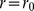 for observers moving along orbits of constant 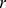 [44]. This then requires 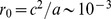 m for the CMB temperature scale, which clearly makes no sense since we would be millimeters away from the center of our observable universe. Nevertheless, the analysis used to derive the Unruh effect implicitly operates under the assumption of the validity of special relativity in flat space-time and therefore takes 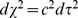. Yet, as we have emphasized repeatedly above, in our theory of inertial centers, the invariant interval associated with the metric is given in terms of proper time by 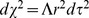. Therefore, we must extend these ideas to apply to our model where we are observers existing within multiple inertial systems (universe 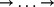 Local Group 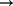 Milky Way). In addition, for our situation, this radiation would not be interpreted physically as due to the “acceleration” of the observer as in the case of [58], but instead one would have to think of this effect as simply the result of the restriction of the Minkowski vacuum to each of the radial Rindler wedges (see Chapters 4.5 and 5.1 of [43]). We are still encouraged that this course of action may result in a plausible interpretation as experimental evidence of large-scale temperature anomalies appears to suggest a significant orientation to the CMB [61].

At this point in our discussion, we offer a brief review of the literature concerning both the Pioneer anomaly as well as the other known astrometric anomalies within our own solar system. First, however, we mention other theories which contrast with our own study but are relevant for the discussion below. The authors of [62] and [63] investigate the potential effects of an expanding universe which could be induced on objects within our solar system. Furthermore, [64] attempts to model the consequences of an extra radial acceleration on the orbital motion of a planet within our solar system. As well, [65] provides an alternative model for gravitation resulting in an additional “Rindler-like” term at large distances which the author claims can potentially model the plateauing nature of observed orbital velocity curves. We must stress that the model proposed in [65] is in fact very different from the model that we have proposed above as our theory of inertial centers does *not* attempt to reformulate gravity. As we emphasized earlier, our model is an attempt to reformulate the motion of objects when no net external forces are acting upon said objects in empty flat space-time. Nevertheless, [66], [67], [68], [69], and [70] use these ideas of an additional “Rindler-like” term in gravitation to examine the possible observable effects of the aforementioned extension to general relativity. For a background reference concerning phenomenology in the context of general relativity, we refer the reader to [71] as preparation for our presentation of the known anomalies exhibited within our own solar system.

Besides the Pioneer anomaly, there are experimental claims of possible anomalies alluding to inconsistencies with our current model for the Solar System. These include:

1. An anomalous secular increase in the eccentricity of the orbit of the Moon
2. The “flyby” anomaly
3. An anomalous correction to the precession of the orbit of Saturn
4. A secular variation of the gravitational parameter 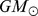 where 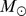 is the mass of the Sun
5. A secular variation of the astronomical unit (AU)

The anomalous secular increase in the eccentricity of the orbit of the Moon was originally found in the experimental analysis of the Lunar Laser Ranging (LLR) data in [72] and expanded upon in [73], [74], [75], and [76]. The “flyby” anomaly refers to an anomalous shift in the Doppler residuals received from spacecrafts when comparing signals before and after these spacecrafts undergo gravitational assists about planets within the Solar System [40] [77] [78]. The anomalous perihelion precession of Saturn appears to be a more controversial claim as the work of [79] and [80] seems to suggest the validity of this observation with further investigation in [81] and [82]. However, work such as [83], [84], and [85] seems to show that this reported anomaly is an experimental artifact. Finally, the last two anomalies of a secular variation in the product of the mass of our Sun and the gravitational constant 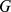 as well as the astronomical unit are more difficult claims to understand in the context of our model as there are many complex mechanisms which could affect our measurements of these quantities (e.g. rate of mass accretion of the Sun from infalling objects versus depletion through expelled radiation resulting from nuclear fusion) in addition to the fact that our measurement of the AU is implicitly linked to our measurement of 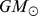 [86]. Nevertheless, [86] and [87] are useful references for these anomalies. Additionally, [23] provides a detailed summary of the majority of the anomalies listed above.

Returning to the Pioneer anomaly, the reader may have concerns with our earlier analysis as recent simulations such as [41] suggest that this anomaly should be taken as a thermal effect from the spacecraft itself instead of evidence linked to “new physics”. For a selection of work concerning the possible thermal explanation of the Pioneer anomaly, see [41], [88], [89], [90], [91], [92], and [93]. Nevertheless, this analysis still does not address the asymmetric nature of the “flyby” anomaly [40] [77] as well as the other significant astrometric Solar System anomalies summarized in [23]. By “asymmetric nature”, we are referring to the fact that the magnitude of the “flyby” anomaly appears to depend upon the direction of approach of the space probe toward Earth as well as the angle of deflection away after “flyby”. Furthermore, as mentioned in [94], the “onset” of the Pioneer anomaly after Pioneer 11's encounter with Saturn is still of concern when explaining these observables as the result of systemic thermal effects. While [41] briefly addresses this “onset” in their conclusion, future analysis of the early data points for Pioneer 11 near its gravitational assist about Saturn appears to be of the utmost importance, especially considering before its encounter with Saturn this spacecraft moved nearly tangentially to the direction of Sagittarius A*, whereas after it traveled nearly toward the Milky Way center. Thus, in the context of our own model, this “onset” has the potential to be interpreted as the consequence of the spacecraft's *change in direction relative to the inertial center associated with the center of the Milky Way*, similar to ideas we will have to explore for the asymmetric nature of the Earth “flyby” anomalies (for potential connections between the Pioneer and “flyby” anomalies, see [40]). Therefore, we choose not to rule out the possibility that the Pioneer anomaly may be support for our theory of inertial centers as this effect as modeled in our earlier analysis in fact must be observed in order for our theory to have physical relevance. As mentioned earlier, we will have to address in far more rigorous detail in future work the dual nature of the Pioneer residuals in order to possibly explain the blueshifts from both Pioneer 10 and Pioneer 11 data.

In addition, others such as [65], [66], and [67] have used a “Rindler-like force” emanating from the center of a gravitational source to supplement general relativistic gravity as a model that can potentially explain orbital velocity curves as well as the Pioneer anomaly [68] [69]. For a review of how this and other gravitational supplements would impact current expectations for the orbits of other major bodies in the Solar System, see [95], [79], [96], [97], [98], [99], [100], [101], [102], [103], [67], [104], [105], [106], [107], [108], and [109]. However, these supplements all require spherical symmetry about the center of the gravitational source in question and are *very different* from our reformulation of flat space-time where in our theory we do *not* assume that there exists a gravitational source at the center of galaxies, groups, clusters, etc. Recall that we are concerned with reformulating inertial motion and inertial reference frames in flat space-time (i.e. our description of the way in which objects move in *flat* space-time when subjected to no net external forces). Additionally, we maintain that locally within confined regions of the inertial system of our theory of inertial centers Einstein's version of gravitation seen as the consequence of space-time curvature induced by the energy-momentum of a massive object in his theory of general relativity still applies in the same manner. In other words, in our theory of inertial centers, this observed deviation from assumed special relativistic flat space-time geodesics arises from our redefinition of the inertial system itself instead of some modification to gravitation. Consequently, when attempting to explain these astrometric Solar System anomalies in the context of our theory, we focus on the difference in geodesics in the galactic inertial reference frame when compared to assumed special relativistic geodesics for flat space-time and assume that *all* of the objects in our Solar System including the Sun orbit about the inertial center point associated with the center of the Milky Way (again, we assume that there is no gravitational source at the center of our galaxy). Meaning, the Pioneer anomaly is not taken to be a phenomenon due to gravity in the theory of inertial centers. Instead the Pioneer anomaly and possibly the other astrometric Solar System anomalies which we have listed above are taken to be the result of our redefinition of inertial systems as well as the change in our expectations for what constitutes inertial motion. Consequently, the relative acceleration between massive objects in our solar system is nearly unchanged from what one would expect from general relativity as all objects within our solar system orbit about the center of the Milky Way along relatively similar paths. Therefore, we are not modifying our expectations for the interactions between objects within the Solar System. We are modifying our expectations for the paths of all objects in the Solar System through the Milky Way. While internally within our solar system the planets remain nearly unchanged in their paths as they move slowly in the “Newtonian limit” (i.e. their speeds are much less than that of light), light propagating between these massive objects in our theory won't behave as one would expect from general relativity as at these speeds one must take into account the properties of the larger inertial system associated with our galaxy.

One must bear in mind that these anomalies are linked to the propagation of electromagnetic radiation throughout our solar system as our experimental apparatuses use light for precision measurements. While the work of [62] attributes the Pioneer anomaly to the local effects of light signal propagation in an expanding universe as expressed by a “post-Friedmannian” metric decomposition, these claims would not be able to explain the asymmetric nature of the wavelength shift residuals in the “flyby” anomaly as the FLRW metric requires homogeneous and isotropic expansion of space in all directions [31]. However, there is *no* expansion in our theory of inertial centers and our inertial reference frames *do* have an orientation. Therefore, we must take into consideration, when comparing with our own model in future work, two important ideas: in this theory of inertial centers, the speed of light is not constant in flat space-time and objects follow inertial paths described by geodesics about inertial centers in the radial Rindler chart, where we assume that the inertial center associated with the Milky Way is in the direction of Sagittarius A*. Thus, in our model, the observables associated with the astrometric Solar System anomalies listed above do not necessarily reflect the existence of an additional acceleration in the Solar System since our theory's radial acceleration would be imposed on *all* objects within the Solar System *including the Sun* and in the same direction toward the center of the Milky Way (10) with seemingly negligible difference in magnitude depending upon the position of the massive object in question (i.e. changes in position within our solar system are negligible relative to the distance of our solar system from the center of the Milky Way when considering the motion of massive satellites, planets, etc.). In other words, in sharp contrast with the analysis in papers such as [65], [109], [97] and [95], we assume that there is no additional acceleration associated with the Sun's gravitational pull on other objects within the Solar System, and thus the relative acceleration of a satellite, planet, etc. with respect to the center of the Sun remains nearly unaffected in our model when we compare with general relativity. Instead, it appears that in the theory of inertial centers these anomalies should more likely be interpreted as a consequence of the non-constant nature of the speed of light within our galactic inertial system as well as of the expected shifts in wavelength when light propagates between differing distances from an inertial center point. Future experiments within the vicinity of our solar system to test the validity of the theory of inertial centers could include sending a spacecraft to the outer edges of our solar system along a closed orbit about the Sun or using identical spacecrafts along open orbits in different directions with respect to the galactic center (e.g. one travels tangentially to the direction of the center of the Milky Way while another moves directly toward/away from the center; for a hyperbolic orbit proposal, see [110]). To test the positional dependence aspects for electromagnetic radiation in this theory, these hypothetical missions should measure the potential variations in wavelength shift and time delay for light signals sent and received at different positions along these orbits with respect to the center of the Milky Way. As well, future theoretical work will require us to explicitly detail observational effects on our astrometric measurements of the planetary ephemerides that are unique to the theory of inertial centers. One could then potentially find these predicted deviations from current models when comparing with the experimental work of [83] and [85].

Using the measured value for the speed of light on Earth (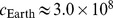 m/s) and the value for the time-scale given from the “time acceleration” in [40], we find that our distance to the center of the Milky Way is approximately 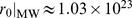 km. We see that the value obtained for our galactic radial distance is far larger than the predicted value from models requiring a supermassive black hole at the center of the Milky Way (intimidatingly, nearly six orders of magnitude [111]). It is imperative then that we reconcile this calculated value with observational data. Not only will this maintain consistency with experiment but it will also provide accurate distance scales within our galaxy. This will allow us to further understand the large observed wavelength shifts near Sagittarius A* within the framework of our theory of inertial centers and potentially explain the paradox of youth [112] through concrete analysis of star formation near the Milky Way center.

Addressing our classical inertial motion analysis, one can immediately tell from the theoretical approach in our discussion that this paper is limited by the lack of necessary quantitative comparison with orbital velocity curves, redshift surveys, and lensing observations. Future work will require modeling using computer simulations of our equations of motion not only to produce orbital velocity curves that will facilitate comparison with data but to also give us a far more thorough understanding of classical inertial motion outside of the limiting behavior examined in this paper. To implement, it appears that we should use a finite difference method with the component form of our geodesic equation parametrized in terms of the proper time of the object in question within a particular inertial system as expanded upon at the end of Appendix B in Appendix S1. Furthermore, we will have to apply this same finite difference method to our normalization condition for the ‘four-velocity’ but parametrized in terms of the proper time in this inertial frame. We also have to attend to a pressing issue with regard to the “Hubble behavior” associated with wavelength shifts within our inertial system. As outlined earlier, this theory requires that we observe *both* significant redshifts and blueshifts, yet on scales larger than the Local Group, blueshifted emitters are reportedly scarce. Thus, if our theory is to be considered seriously, we must provide an explanation for why there is such an imbalance towards reported redshifted emitters at the largest observable scales. Nevertheless, one apparent resolution lies in the possible alternative “blueshift interpretation” of spectroscopic profiles as mentioned and subsequently applied in [113], [114], and [115] with possible support for the re-examination of spectroscopic profiles in the blueshifted emission lines found in other work such as [116].

Proceeding to our quantum concerns, our seemingly shocking proposal that at the center of the nucleus of every atom there could potentially exist an inertial center point raises many more questions for our theory of inertial centers. Of course, this type of claim requires thorough and rigorous justification in both future theoretical work and even more importantly in comparison with experiment. For example, a simple comparison with experiment would be to determine how accurate of a fit our “n-particle amplitudes” (reviewed in Appendix D in Appendix S1) with individual solutions for quantum numbers 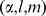 given by (64) are with current experimental knowledge of the nucleus. Nevertheless, we have chosen to mention these ideas in this paper in order to highlight to the reader how much of a potential impact this redefinition of inertial motion and inertial reference frames could possibly have on our understanding of structure formation for all scales from the largest to the smallest. As for questions: for one, can we reconcile these claims with our current knowledge of the electronic and nuclear structure of the atom when we factor in charge, spin, and electromagnetism? Additionally, how much of our current model for the nucleus is affected by these ideas? It also becomes ever more important to answer the following: What establishes one of these inertial centers as well as the orientation of one of our inertial systems?

## Conclusions

All of our assumptions within this work in one way or another are built upon the idea that objects do *not* move in a straight line at a constant speed when no external forces are acting upon them in empty flat space-time. In other words, we assume that Newton's first law does not give the correct characterization of inertial motion. Therefore, we essentially “start from scratch” and concentrate on how to incorporate all of the following observed features into a revised understanding of inertial motion: accelerated redshifts and the Hubble relation, plateauing orbital velocity curves at large distances from a central point about which objects move, consistent velocity “flow” on the largest of scales directed toward a central point, and an orientation associated with each of these central points. We take an inertial frame of reference to be the system within which objects follow these revised inertial trajectories and begin our reformulation with the knowledge that our theory of globally flat space-time must reduce to special relativity within confined regions of our newly defined inertial systems. Consequently, it appears natural to approach this reformulation from the notion that we should have a metric theory of flat space-time, and within this metric theory objects still follow along geodesic trajectories when no external forces are acting upon them as in special and general relativity. However, in order to distinguish our metric theory of flat space-time from special relativity, we must require that our affine parameter *not* be proper time globally throughout these reference frames. In addition, we find that we are able to reproduce the previously listed features with the radial Rindler chart as the coordinate parametrization of our flat space-time manifold, thereby assuming the physical significance of special central points which we deem “inertial center points” situated throughout all of space-time. As one would expect from their given name, these inertial center points describe the centers of each of our inertial systems, and our inertial trajectories are then assumed to be the orbits of objects about these inertial centers. Meaning, inertial motion must be thought of relative to both the center point and the orientation (i.e. location of the poles) of each of these inertial reference frames. Consequently, it is assumed that the observed motion of objects about central points on the largest of scales (e.g. stars orbiting the center of a galaxy, galaxies orbiting the center of a group/cluster, etc.) is *not* due to gravitational effects but is instead a manifestation of inertial motion within our theory of flat space-time, which we term our “Theory of Inertial Centers”.

This redefinition of inertial motion then allows us to no longer assume the existence of ‘dark energy’, ‘dark matter’, and ‘dark flow’. Furthermore, as we have the ability to model the Hubble relation within our theory, we do not require the occurrence of a ‘Big-Bang’ event, and therefore we also do not require ‘inflation’ nor an expanding universe (i.e. we do not operate under the assumptions of 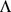
CDM). The cornerstone of our theory is embodied in the statement that within our inertial systems, time and space are fundamentally intertwined such that time- and spatial-translational invariance are *not* inherent symmetries of flat space-time. Meaning, our invariant interval associated with the metric incorporates *both* time and spatial distance. Therefore, observable clock rates depend upon not only the relative velocity of observers within these inertial systems but also on the difference in distance of each observer from an inertial center, expressed mathematically by relation (57). Given this relation, we find that our theory of globally flat space-time in fact reduces to special relativity for observers which we can consider as nearly stationary with respect to the inertial center point about which they orbit (i.e. the local stationary limit). As well, our ideas then require that the local speed of light which we measure within a confined region of these newly defined inertial systems is linearly dependent upon our distance away from the inertial center about which we orbit (56). Thus, the speed of light throughout each of these redefined inertial systems in flat space-time is not constant.

With these theoretical foundations presented, we proceeded by examining the local consequences of our theory for a gravitational system located within one of these inertial systems as an observer should be able to measure with a detector of the necessary sensitivity the deviation of an object's (specifically light's) inertial path in flat space-time away from special relativistic geodesics and into the geodesics of our theory as outlined in the local stationary limit. Thus, within the framework of the theory of inertial centers, we interpret the Pioneer anomaly as an observable consequence of our revised ideas on inertial motion. However, as mentioned later in our paper, there are many open questions that must be answered with regard to the propagation of light signals within our solar system in the context of our theory. Specifically, can our revision of inertial motion and inertial reference frames explain the other known astrometric Solar System anomalies (i.e. “flyby” anomaly, the anomalous increase in the eccentricity of the Moon, and the variation in the AU)? And, can we explain the blueshifted nature of both Pioneer 10 and Pioneer 11 Doppler data once we factor in the two-way nature of these residuals as well as the change in clock rates for observers located at different distances from the center of the Milky Way in our model?

Furthermore, after quantizing for a real massive scalar field, we came upon a potential explanation for the asymmetry between matter and antimatter in our observable universe within the context of our theory of inertial centers. If we allow for the possibility that our field exists in both radial Rindler wedges (i.e. 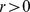 and 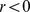), it appears that a logical explanation for the observable imbalance toward matter would be that our antimatter counterparts are located in the “other” radial Rindler wedge for each of our inertial systems, as the charge of each field in these systems should be conserved (e.g. abundance of electrons in one wedge should imply an abundance of positrons in the “other” wedge). Nevertheless, this logic relies on the consistency of our extension for a real scalar field to complex fields with spin. Thus, in future work, we will have to address the validity of this interpretation when we extend our analysis (e.g. Dirac spinors). In addition, we concluded our discussion by examining the nearly stationary limit for particles close to an inertial center point. Using expression (10), we chose to work naively under Newton's assumptions and take this acceleration on our observer to be the result of a Newtonian force derived from a conservative potential. Then, the stationary Hamiltonian associated with this Newtonian approximation would take the form of the isotropic harmonic oscillator. Taking the perspective of an observer exterior to the inertial system in question (i.e. the external observer orbits a *different* inertial center), we found the observed oscillator energy scale using relation (57) while operating under the assumption that the time-scale for each inertial system is a universal constant and therefore the same for each. A simple potential explanation for the ability of the isotropic harmonic oscillator to explain the “magic numbers” associated with stable arrangements of nucleons within the nucleus of an atom then arose in the context of our model. Since both the form of our stationary Hamiltonian as well as the determined energy scale match that of the starting point for our nuclear shell models, it appears that we must seriously consider the possibility that there exists an inertial center point at the center of the nucleus of every atom when working under the assumptions of our theory of inertial centers as, in our stationary limit, the acceleration of each particle within the inertial system mimics what one would find if he/she naively assumed a Newtonian Hamiltonian of the form of the isotropic harmonic oscillator. In other words, within the context of our theory, the ability of the isotropic harmonic oscillator to model the simplest nuclear configurations would be interpreted as a consequence of the physical existence of an inertial center located at the center of the nucleus of every atom, where these simple configurations of nucleons arise from the stationary limit for objects very near to an inertial center. Although these claims are radical in nature, we are still compelled to question whether or not the nuclear ‘force’ is even really a force within the framework of our model. Future theoretical and experimental work will be required in order to fully understand the nature of these ideas.

## Supporting Information

Appendix S1 (PDF)
