# Correction: Re-Examination of Globally Flat Space-Time

**DOI:** 10.1371/annotation/41868a14-4583-48e7-8fde-499055b2ec69

**Published:** 2013-12-17

**Authors:** Michael R. Feldman

The ϑ symbol erroneously appeared in Equations, 2, 6, 26, 29, 63, 65 and in the limit expression above Equation 40. All occurrences of ϑ should be replaced by the φ symbol.

The Laplace-Belmatri operator in Equation 60 and the following sentence should be represented by a tombstone symbol □ instead of a W.

The second expression after the sentence "Our metric equation takes the form of the Schwarzschild solution..." in the Discussion section also erroneously contains the ϑ symbol. The φ symbol should appear in its place.

Please see the correct equations here: http://www.plosone.org/corrections/pone.0078114.001.cn.tif 

