# Supplementary material for: Re-Examination of Globally Flat Space-Time
Source: PLoS One. 2013 Nov 8;8(11):e78114. doi: 10.1371/journal.pone.0078114 (PMC3826743; doi:10.1371/journal.pone.0078114)
Supplement: Appendix S1 — (PDF) [file pone.0078114.s001.pdf]

A Affine connection terms, Ricci and Riemann tensors

Following [24] for our general expressions below, we work in the metric

$$ds^2 = -\Lambda r^2 dt^2 + dr^2 + r^2 \cosh^2(\sqrt{\Lambda}t)[d\theta^2 + d\phi^2 \sin^2\theta]$$

Our affine connection tensor for this choice of coordinates is given by the expression:

$$\Gamma^c_{ab} = \frac{1}{2} g^{cd}[\partial_a g_{db} + \partial_b g_{da} - \partial_d g_{ab}]$$

where in component form $\partial_\mu = \partial/\partial x^\mu$ is our ordinary partial derivative and in radial Rindler coordinates our metric components gathered in matrix form are given by

$$(g_{\mu\nu}) = \begin{pmatrix} -\Lambda r^2 & 0 & 0 & 0 \\ 0 & 1 & 0 & 0 \\ 0 & 0 & r^2\cosh^2(\sqrt{\Lambda}t) & 0 \\ 0 & 0 & 0 & r^2\cosh^2(\sqrt{\Lambda}t)\sin^2\theta \end{pmatrix} \tag{A.1}$$

$$(g^{\mu\nu}) = \begin{pmatrix} -\frac{1}{\Lambda r^2} & 0 & 0 & 0 \\ 0 & 1 & 0 & 0 \\ 0 & 0 & \frac{1}{r^2\cosh^2(\sqrt{\Lambda}t)} & 0 \\ 0 & 0 & 0 & \frac{1}{r^2\cosh^2(\sqrt{\Lambda}t)\sin^2\theta} \end{pmatrix} \tag{A.2}$$

with $(g^{\mu\nu})$ corresponding to the inverse of the matrix associated with $(g_{\mu\nu})$ $(g_{tt} = -\Lambda r^2, g_{rr} = 1, g_{\theta\theta} = r^2\cosh^2(\sqrt{\Lambda}t), g_{\phi\phi} = r^2\cosh^2(\sqrt{\Lambda}t)\sin^2\theta)$. For the reader who may be unfamiliar with abstract index notation, we look for each affine connection term associated with the tensor above by examining this expression in component form:

$$\Gamma^\lambda_{\mu\nu} = \frac{1}{2}\sum_\rho g^{\lambda\rho}[\partial_\mu g_{\rho\nu} + \partial_\nu g_{\rho\mu} - \partial_\rho g_{\mu\nu}]$$

where we use greek indices (e.g. λ, μ, ν) for components and latin indices (e.g. a, b, c) for the tensor itself. Just as an example, we look for the component Γ^t_{tr}:

$$\Gamma^t_{tr} = \frac{1}{2}\sum_\beta g^{t\beta}[\partial_t g_{\beta r} + \partial_r g_{\beta t} - \partial_\beta g_{tr}]$$

where we sum over like indices for $\beta = t,\ r,\ \theta,\ \phi$. Then,

$$\Gamma^t_{tr} = \frac{1}{2}\Big\{ g^{tt}[\partial_t g_{tr} + \partial_r g_{tt} - \partial_t g_{tr}] + g^{tr}[\partial_t g_{rr} + \partial_r g_{rt} - \partial_r g_{tr}] + g^{t\theta}[\partial_t g_{\theta r} + \partial_r g_{\theta t} - \partial_\theta g_{tr}] + g^{t\phi}[\partial_t g_{\phi r} + \partial_r g_{\phi t} - \partial_\phi g_{tr}] \Big\}$$

However, from (A.2), we know that $g^{tr} = g^{t\theta} = g^{t\phi} = 0$. Therefore, our expression reduces to

$$\Gamma^t_{tr} = \frac{1}{2} g^{tt}[\partial_t g_{tr} + \partial_r g_{tt} - \partial_t g_{tr}]$$

Yet from (A.1), we see that $g_{tr} = 0$. This leaves us with

$$\Gamma^t_{tr} = \frac{1}{2} g^{tt}\partial_r g_{tt} = \frac{1}{2}\Big(-\frac{1}{\Lambda r^2}\Big)\cdot \partial_r(-\Lambda r^2) = \frac{1}{r}$$

For the reader who wishes to derive the rest of these affine connection components, we notice from (A.1) that $g_{\mu\nu} = 0$ for $\mu \neq \nu$ (our metric is diagonal in radial Rindler coordinates). Then the expression for our affine connection terms reduces to

$$\Gamma^{\lambda}_{\mu\nu} = \frac{1}{2} g^{\lambda\lambda} [\partial_\mu g_{\lambda\nu} + \partial_\nu g_{\lambda\mu} - \partial_\lambda g_{\mu\nu}]$$

Yet, because Γ^c_{ab} is symmetric in $a \Leftrightarrow b$ (i.e. if we swap a and b indices, our tensor remains the same as one can see above since g_{ab} is also symmetric under the same exchange by definition), our possibilities for the affine connection terms are limited to three cases: $\lambda = \nu \neq \mu$; $\lambda = \nu = \mu$; $\mu = \nu \neq \lambda$. For $\lambda = \nu \neq \mu$,

$$\Gamma^{\lambda}_{\mu\lambda} = \frac{1}{2} g^{\lambda\lambda} [\partial_\mu g_{\lambda\lambda} + \partial_\lambda g_{\lambda\mu} - \partial_\lambda g_{\mu\lambda}] = \frac{1}{2} g^{\lambda\lambda} \partial_\mu g_{\lambda\lambda} \qquad (\lambda \neq \mu)$$

where we used the diagonal property of our metric parametrization in the last equality. Applying similar logic to our other two cases, we obtain

$$\Gamma^{\lambda}_{\lambda\lambda} = \frac{1}{2} g^{\lambda\lambda} \partial_\lambda g_{\lambda\lambda} \quad \text{and} \quad \Gamma^{\lambda}_{\mu\mu} = -\frac{1}{2} g^{\lambda\lambda} \partial_\lambda g_{\mu\mu} \qquad (\lambda \neq \mu)$$

Using these identities, one finds that our non-zero affine connection terms are:

$$\Gamma^t_{tr} = \frac{1}{r} \qquad \Gamma^t_{\theta\theta} = \frac{1}{\sqrt{\Lambda}} \cosh(\sqrt{\Lambda} t) \sinh(\sqrt{\Lambda} t) \qquad \Gamma^t_{\phi\phi} = \frac{1}{\sqrt{\Lambda}} \cosh(\sqrt{\Lambda} t) \sinh(\sqrt{\Lambda} t) \sin^2\theta$$
$$\Gamma^r_{tt} = \Lambda r \qquad \Gamma^r_{\theta\theta} = -r \cosh^2(\sqrt{\Lambda} t) \qquad \Gamma^r_{\phi\phi} = -r \cosh^2(\sqrt{\Lambda} t) \sin^2\theta$$
$$\Gamma^\theta_{\theta t} = \Gamma^\phi_{\phi t} = \sqrt{\Lambda} \tanh(\sqrt{\Lambda} t) \qquad \Gamma^\theta_{\theta r} = \Gamma^\phi_{\phi r} = \frac{1}{r} \qquad \Gamma^\theta_{\phi\phi} = -\sin\theta\cos\theta \qquad \Gamma^\phi_{\phi\theta} = \cot\theta$$

We define the curvature tensor by the action of the linear map $(\nabla_a \nabla_b - \nabla_b \nabla_a)$ on a dual vector field ω_c (for more information on vector fields, see Chapter 2 of [24]):

$$\nabla_a \nabla_b \omega_c - \nabla_b \nabla_a \omega_c = R_{abc}{}^d \omega_d$$

where ∇_a is the derivative operator compatible with our metric (or covariant derivative; $\nabla_a g_{bc} = 0$) and we refer to $R_{abc}{}^d$ as the Riemann curvature tensor. Our Riemann curvature tensor can be expressed in terms of the affine connection associated with a particular choice of coordinate chart:

$$R_{abc}{}^d = \partial_b \Gamma^d_{ac} - \partial_a \Gamma^d_{bc} + \Gamma^e_{ca} \Gamma^d_{be} - \Gamma^e_{cb} \Gamma^d_{ae}$$

And in component form,

$$R_{\mu\nu\rho}{}^\sigma = \partial_\nu \Gamma^\sigma_{\mu\rho} - \partial_\mu \Gamma^\sigma_{\nu\rho} + \sum_\lambda \left[\Gamma^\lambda_{\rho\mu} \Gamma^\sigma_{\nu\lambda} - \Gamma^\lambda_{\rho\nu} \Gamma^\sigma_{\mu\lambda} \right]$$

Using our affine connection terms, we discover $R_{\mu\nu\rho}{}^\sigma = 0 \ \forall \mu, \nu, \rho, \sigma$ as we should expect since the radial Rindler chart is just a coordinate transformation away from the Minkowski chart (the geometric properties of the manifold are independent of coordinate parametrization). Therefore, $R_{\mu\beta} = \sum_\lambda R_{\mu\lambda\beta}{}^\lambda = 0$ and $\sum_{\lambda,\mu,\nu,\beta} R^{\lambda\mu\nu\beta} R_{\lambda\mu\nu\beta} = 0$. The metric satisfies the Einstein field equations in vacuum without a cosmological constant [6] and represents flat space-time.

B Equations of motion

$$0 = U^a \nabla_a U^b \tag{B.1}$$

where ∇_a is the derivative operator compatible with our metric (or covariant derivative; $\nabla_a g_{bc} = 0$). For the reader who may be unfamiliar with concepts in differential geometry, the action of this derivative operator on an arbitrary vector field (a vector field is an assignment of a vector at each point on the manifold) can be expressed in terms of our more familiar partial derivatives through the affine connection tensor associated with a particular coordinate system. When our derivative operator acts on an arbitrary vector field v^a, we have

$$\nabla_a v^b = \partial_a v^b + \Gamma^b_{ac} v^c$$

and for this same derivative operator acting upon a dual vector field,

$$\nabla_a v_b = \partial_a v_b - \Gamma^c_{ab} v_c$$

Without going into further detail with regard to vector spaces, the reader may feel more informed to know that we can relate a vector with its dual space counterpart through the metric:

$$v_a = g_{ab} v^b$$

In addition, a vector v^a given at each point on a curve C is said to be parallelly transported as one moves along this curve if

$$t^a \nabla_a v^b = 0$$

where t^a refers to the tangent vector to the curve. We then define a geodesic to be a curve whose tangent denoted U^a satisfies (B.1) (for more on parallel transport, see Chapter 3.3 of [24]) and assume that our particles travel along these curves when subjected to no net external forces. Additionally, a parametrization of a curve which yields (B.1) is called an affine parametrization, and thus by definition a geodesic is required to be affinely parametrized.

For a geodesic along which one of our particles moves denoted $x^\mu(\sigma)$ in our particular coordinate system and parametrized in terms of the affine parameter σ, our tangent vector to this curve in component form is given by $U^\mu = dx^\mu/d\sigma$ (where $\sigma = \chi$ for massive particles) and is said to be the 'proper velocity' or 'four-velocity' of this particle. We also define

$$\chi = \int (-g_{ab} T^a T^b)^{1/2} dt$$

where T^a is the tangent vector to any particular time-like (i.e. $g_{ab} T^a T^b < 0$) curve and t is an arbitrary parametrization of this curve. Thus, along a time-like geodesic affinely parameterized by χ, we have

$$g_{ab} U^a U^b = -1$$

Applying all of the above concepts to expand our equation of motion (B.1) in a particular coordinate system,

$$0 = U^a \Big[\partial_a U^b + \Gamma^b_{ac} U^c \Big]$$

Using our expression for the 'proper velocity' in component form, we come upon the geodesic equation of motion for particles in terms of our affine connection terms:

$$\begin{aligned} 0 &= \sum_\alpha \frac{dx^\alpha}{d\sigma} \cdot \frac{\partial}{\partial x^\alpha} \left(\frac{dx^\nu}{d\sigma}\right) + \sum_{\mu,\rho} \Gamma^\nu_{\mu\rho} \frac{dx^\mu}{d\sigma} \frac{dx^\rho}{d\sigma} \\ &= \frac{d^2 x^\nu}{d\sigma^2} + \sum_{\mu,\rho} \Gamma^\nu_{\mu\rho} \frac{dx^\mu}{d\sigma} \frac{dx^\rho}{d\sigma} \end{aligned}$$

Therefore, for our radial Rindler metric, we can plug in the affine connection terms found in Appendix A where in addition $x^\mu(\sigma) \to \langle t(\sigma), r(\sigma), \theta(\sigma), \phi(\sigma)\rangle$. This notation for our vector components signifies

$$x^a = t(\sigma)\left(\frac{\partial}{\partial t}\right)^a + r(\sigma)\left(\frac{\partial}{\partial r}\right)^a + \theta(\sigma)\left(\frac{\partial}{\partial \theta}\right)^a + \phi(\sigma)\left(\frac{\partial}{\partial \phi}\right)^a$$

where $(\partial/\partial t)^a$, $(\partial/\partial r)^a$, $(\partial/\partial\theta)^a$, and $(\partial/\partial\phi)^a$ are linearly independent tangent vectors which span the tangent spaces at each point on the manifold. For example, we take our equation of motion for $t(\sigma)$:

$$\begin{aligned} 0 &= \frac{d^2t}{d\sigma^2} + \sum_{\mu,\rho} \Gamma^t_{\mu\rho}\frac{dx^\mu}{d\sigma}\frac{dx^\rho}{d\sigma} \\ &= \frac{d^2t}{d\sigma^2} + \Gamma^t_{tr}\frac{dt}{d\sigma}\frac{dr}{d\sigma} + \Gamma^t_{rt}\frac{dr}{d\sigma}\frac{dt}{d\sigma} + \Gamma^t_{\theta\theta}\frac{d\theta}{d\sigma}\frac{d\theta}{d\sigma} + \Gamma^t_{\phi\phi}\frac{d\phi}{d\sigma}\frac{d\phi}{d\sigma} \end{aligned}$$

However, we know from our work in Appendix A that $\Gamma^t_{tr} = \Gamma^t_{rt}$. Consequently, we find after plugging in for each affine connection term

$$0 = \frac{d^2t}{d\sigma^2} + \frac{2}{r}\frac{dt}{d\sigma}\frac{dr}{d\sigma} + \frac{1}{\sqrt{\Lambda}}\cosh(\sqrt{\Lambda}t)\sinh(\sqrt{\Lambda}t)\left[\left(\frac{d\theta}{d\sigma}\right)^2 + \sin^2\theta\left(\frac{d\phi}{d\sigma}\right)^2\right]$$

Applying similar logic for $\nu = r$, θ, and ϕ:

$$\begin{aligned} 0 &= \frac{d^2r}{d\sigma^2} + \Lambda r\left(\frac{dt}{d\sigma}\right)^2 - r\cosh^2(\sqrt{\Lambda}t)\left[\left(\frac{d\theta}{d\sigma}\right)^2 + \sin^2\theta\left(\frac{d\phi}{d\sigma}\right)^2\right] \\ 0 &= \frac{d^2\theta}{d\sigma^2} + 2\frac{d\theta}{d\sigma}\left[\sqrt{\Lambda}\tanh(\sqrt{\Lambda}t)\frac{dt}{d\sigma} + \frac{1}{r}\frac{dr}{d\sigma}\right] - \sin\theta\cos\theta\left(\frac{d\phi}{d\sigma}\right)^2 \\ 0 &= \frac{d^2\phi}{d\sigma^2} + 2\frac{d\phi}{d\sigma}\left[\sqrt{\Lambda}\tanh(\sqrt{\Lambda}t)\frac{dt}{d\sigma} + \frac{1}{r}\frac{dr}{d\sigma} + \cot\theta\frac{d\theta}{d\sigma}\right] \end{aligned}$$

As briefly mentioned above, if one evaluates the norm of the 'proper velocity', he/she will find:

$$U^aU_a = \sum_{\mu,\nu} g_{\mu\nu}U^\mu U^\nu = \sum_{\mu,\nu} g_{\mu\nu}\frac{dx^\mu}{d\sigma}\frac{dx^\nu}{d\sigma} = \begin{cases} 0 & \text{null geodesics} \\ -1 & \text{time-like geodesics} \end{cases}$$

In a relatively simple way, one can see this from our line element where $-d\chi^2 = \sum_{\mu,\nu} g_{\mu\nu}dx^\mu dx^\nu$. Massless particles travel along null geodesics (i.e. our norm vanishes) whereas massive particles travel along time-like geodesics. For comparison with special relativity and general relativity, we express the component form of our time-like geodesics where $\sigma = \chi$ in terms of the physically observable elapsed time as measured by a clock carried along the given curve in a particular inertial system, τ:

$$0 = \frac{d^2\tau}{d\chi^2}\frac{dx^\nu}{d\tau} + \left(\frac{d\tau}{d\chi}\right)^2 \cdot \left[\frac{d^2x^\nu}{d\tau^2} + \sum_{\mu,\rho}\Gamma^\nu_{\mu\rho}\frac{dx^\mu}{d\tau}\frac{dx^\rho}{d\tau}\right]$$

One immediately notices that the term in brackets represents the component form of the geodesic equation for special and general relativity and would be set equal to zero in both of these theories. However, since in the theory of inertial centers $d^2\tau/d\chi^2 \neq 0$ as $d\chi/d\tau = \sqrt{\Lambda}\cdot r(\tau)$, the term in brackets is *not* necessarily zero for our theory, and thus the observed inertial motion of massive objects in our model characterized by the equation above is in fact very different from inertial motion as seen in special and general relativity.

C Killing vector fields

As in our previous appendices, we provide a summary of [24] with regard to the more general statements below (see Appendix C and Chapter 2 of [24]). In order to understand the relevance of Killing vector fields with respect to inherent symmetries associated with our manifold, we must begin with a brief introduction to isometries and Lie derivatives. For two manifolds M and N, let ϕ be a smooth map from M to N ($\phi : M \to N$) and f be a function from N to the reals ($f : N \to \mathbb{R}$). Then the composition of f with ϕ, $f \circ \phi$, produces a function from $M \to \mathbb{R}$ and ϕ is said to "pull back" f. In addition ϕ "carries along" tangent vectors at a particular point $p \in M$ to tangent vectors at $\phi(p) \in N$, and therefore defines a map $\phi^\star : V_p \to V_{\phi(p)}$ in the following manner:

$$(\phi^\star v)(f) = v(f \circ \phi)$$

where $v \in V_p$, $\phi^\star v \in V_{\phi(p)}$, and V_p denotes the tangent vector space at p. One can also use ϕ to "pull back" dual vectors at $\phi(p)$ to dual vectors at p by defining a map $\phi_\star : V^\star_{\phi(p)} \to V^\star_p$ requiring for all $v^a \in V_p$

$$(\phi_\star \mu)_a v^a = \mu_a (\phi^\star v)^a$$

where $V^\star_p$ denotes the dual vector space at p. If $\phi : M \to N$ is a diffeomorphism (i.e. a smooth function that is one-to-one, onto, and its inverse is also smooth), then for an arbitrary tensor $T^{b_1 \ldots b_k}{}_{a_1 \ldots a_l}$ of type (k, l) at p (type (k, l) refers to the number of dual vector "slots" and vector "slots", respectively), the tensor $(\phi^\star T)^{b_1 \ldots b_k}{}_{a_1 \ldots a_l}$ at $\phi(p)$ is defined by

$$(\phi^\star T)^{b_1 \ldots b_k}{}_{a_1 \ldots a_l} (\mu_1)_{b_1} \cdots (\mu_k)_{b_k} (t_1)^{a_1} \cdots (t_l)^{a_l} = T^{b_1 \ldots b_k}{}_{a_1 \ldots a_l} (\phi_\star \mu_1)_{b_1} \cdots ([\phi^{-1}]^\star t_l)^{a_l}$$

as $(\phi^{-1})^\star : V_{\phi(p)} \to V_p$. If $\phi : M \to M$ is a diffeomorphism and T is a tensor field on M, then we refer to ϕ as a symmetry transformation for the tensor field T if $\phi^\star T = T$. In addition, if

$$(\phi^\star g)_{ab} = g_{ab}$$

we refer to ϕ as an isometry.

To introduce the notion of Lie derivatives, we come back to diffeomorphisms and define a one-parameter group of diffeomorphisms ϕ_t as a smooth map from $\mathbb{R} \times M \to M$ such that for fixed $t \in \mathbb{R}$, $\phi_t : M \to M$ is a diffeomorphism. As well, for all $t, s \in \mathbb{R}$, $\phi_t \circ \phi_s = \phi_{t+s}$. In particular, this requires $\phi_{t=0}$ to be the identity map. A vector field v^a can be thought of as the infinitessimal generator of a one-parameter group of finite transformations of M in the following manner. For fixed $p \in M$, we refer to the curve $\phi_t(p) : \mathbb{R} \to M$ as an orbit of ϕ_t which passes through p at $t = 0$. $v|_p$ is defined to be the tangent to this curve at $t = 0$. We also define the Lie derivative with respect to v^a by

$$\mathfrak{L}_v T^{b_1 \ldots b_k}{}_{a_1 \ldots a_l} = \lim_{t \to 0} \left\{ \frac{\phi^\star_{-t} T^{b_1 \ldots b_k}{}_{a_1 \ldots a_l} - T^{b_1 \ldots b_k}{}_{a_1 \ldots a_l}}{t} \right\}$$

where all tensors above are evaluated at a point p. $\mathfrak{L}_v$ is then a linear map from smooth tensor fields of type (k, l) to smooth tensor fields of type (k, l) and satisfies the Leibniz rule on outer products of tensors. Since v^a is tangent to the integral curves of ϕ_t, for functions $f : M \to \mathbb{R}$

$$\mathfrak{L}_v(f) = v(f)$$

In addition, if ϕ_t is a symmetry transformation for T, we have $\mathfrak{L}_v T^{b_1 \ldots b_k}{}_{a_1 \ldots a_l} = 0$. Furthermore, it is found that the Lie derivative with respect to v^a of a vector field w^a is given by the commutator:

$$\mathfrak{L}_v w^a = [v, w]^a$$

where

$$[v,w]^a = v^b\nabla_b w^a - w^b\nabla_b v^a$$

and for a dual vector,

$$\mathfrak{L}_v\mu_a = v^b\nabla_b\mu_a + \mu_b\nabla_a v^b$$

The more general action of a Lie derivative with respect to v^a on a general tensor field $T^{b_1\ldots b_k}{}_{a_1\ldots a_l}$ is given by

$$\mathfrak{L}_v T^{b_1\ldots b_k}{}_{a_1\ldots a_l} = v^c\nabla_c T^{b_1\ldots b_k}{}_{a_1\ldots a_l} - \sum_{i=1}^{k} T^{b_1\ldots c\ldots b_k}{}_{a_1\ldots a_l}\nabla_c v^{b_i} + \sum_{j=1}^{l} T^{b_1\ldots b_k}{}_{a_1\ldots c\ldots a_l}\nabla_{a_j} v^c$$

where ∇_a is our derivative operator compatible with the metric g_{ab} (i.e. $\nabla_c g_{ab} = 0$). Then a Killing vector field ξ^a is defined to be the vector field which generates a one-parameter group of isometries $\phi_t : M \to M$ of the metric, $(\phi_t^\star g)_{ab} = g_{ab}$. As remarked earlier, the necessary condition for ϕ_t to be a group of isometries is $\mathfrak{L}_\xi g_{ab} = 0$. Using the expression above for the action of a Lie derivative on a tensor field,

$$\begin{aligned}\mathfrak{L}_\xi g_{ab} &= \xi^c\nabla_c g_{ab} + g_{cb}\nabla_a\xi^c + g_{ac}\nabla_b\xi^c \\ &= \nabla_a\xi_b + \nabla_b\xi_a\end{aligned}$$

Thus, we come upon Killing's equation:

$$\nabla_a\xi_b + \nabla_a\xi_b = 0$$

For any particular Killing vector field ξ^a, along a geodesic γ with tangent vector U^a one finds

$$\begin{aligned}U^b\nabla_b(\xi_a U^a) &= U^b U^a\nabla_b\xi_a + \xi^a U^b\nabla_b U^a \\ &= \frac{1}{2}U^a U^b[\nabla_b\xi_a + \nabla_a\xi_b] + \xi^a U^b\nabla_b U^a = 0\end{aligned}$$

where the first term vanishes by Killing's equation and the second by the geodesic equation (B.1). Meaning, along γ, $\xi^a U_a$ is constant (Noether's theorem).

Using our affine connection component terms, Killing's equation takes the form

$$\partial_\mu\xi_\nu + \partial_\nu\xi_\mu = 2\sum_\rho \Gamma^\rho_{\mu\nu}\xi_\rho$$

which gives for each pair (μ,ν),

$$(t,t) : \partial_t\xi_t = \Lambda r\xi_r \qquad (t,r) : \partial_t\xi_r + \partial_r\xi_t = \frac{2}{r}\xi_t \qquad (t,\theta) : \partial_t\xi_\theta + \partial_\theta\xi_t = 2\sqrt{\Lambda}\tanh(\sqrt{\Lambda}t)\xi_\theta$$

$$(t,\phi) : \partial_t\xi_\phi + \partial_\phi\xi_t = 2\sqrt{\Lambda}\tanh(\sqrt{\Lambda}t)\xi_\phi \qquad (r,r) : \partial_r\xi_r = 0 \qquad (r,\theta) : \partial_\theta\xi_r + \partial_r\xi_\theta = \frac{2}{r}\xi_\theta$$

$$(r,\phi) : \partial_\phi\xi_r + \partial_r\xi_\phi = \frac{2}{r}\xi_\phi \qquad (\theta,\phi) : \partial_\theta\xi_\phi + \partial_\phi\xi_\theta = 2\cot\theta\xi_\phi$$

$$(\theta,\theta) : \partial_\theta\xi_\theta = \frac{1}{\sqrt{\Lambda}}\cosh(\sqrt{\Lambda}t)\sinh(\sqrt{\Lambda}t)\xi_t - r\cosh^2(\sqrt{\Lambda}t)\xi_r$$

$$(\phi,\phi) : \partial_\phi\xi_\phi = \sin^2\theta\left[\frac{1}{\sqrt{\Lambda}}\cosh(\sqrt{\Lambda}t)\sinh(\sqrt{\Lambda}t)\xi_t - r\cosh^2(\sqrt{\Lambda}t)\xi_r\right] - \sin\theta\cos\theta\xi_\theta$$

Immediately we notice from the (t,t) equation that $\xi_t = 0 \Longrightarrow \xi_r = 0$ and from the (θ,θ) equation that $\xi_r = \xi_\theta = 0 \Longrightarrow \xi_t = 0$. For $\xi_t = \xi_r = 0$, we find the three rotational Killing vector fields:

$$\begin{aligned}\Omega_1^\mu &\to \langle 0, 0, \cos\phi, -\cot\theta\sin\phi\rangle\\ \Omega_2^\mu &\to \langle 0, 0, \sin\phi, \cot\theta\cos\phi\rangle\\ \psi^\mu &\to \langle 0,0,0,1\rangle\end{aligned}$$

For $\xi_\theta = \xi_\phi = 0$, we have a time and radial Killing vector field:

$$\rho^\mu \to \langle \frac{1}{\sqrt{\Lambda}r}\cosh(\sqrt{\Lambda}t), -\sinh(\sqrt{\Lambda}t), 0, 0\rangle$$

For $\xi_r = \xi_\phi = 0$, a time and θ Killing vector field:

$$\Theta^\mu \to \langle \frac{1}{\sqrt{\Lambda}}\cos\theta, 0, -\sin\theta\tanh(\sqrt{\Lambda}t), 0\rangle$$

For $\xi_\phi = 0$, a time, radial, and θ Killing vector field:

$$\xi^\mu_{(t,r,\theta)} = \langle -\frac{\sinh(\sqrt{\Lambda}t)\cos\theta}{\sqrt{\Lambda}r}, \cosh(\sqrt{\Lambda}t)\cos\theta, -\frac{\sin\theta}{r\cosh(\sqrt{\Lambda}t)}, 0\rangle$$

In addition, for only $\xi_r = 0$, we find two Killing vector fields:

$$\begin{aligned}\xi^\mu_{(t,\theta,\phi),1} &\to \langle \frac{1}{\Lambda}\sin\theta\sin\phi, 0, \frac{1}{\sqrt{\Lambda}}\tanh(\sqrt{\Lambda}t)\cos\theta\sin\phi, \frac{1}{\sqrt{\Lambda}\sin\theta}\tanh(\sqrt{\Lambda}t)\cos\phi\rangle\\ \xi^\mu_{(t,\theta,\phi),2} &\to \langle -\frac{1}{\Lambda}\sin\theta\cos\phi, 0, -\frac{1}{\sqrt{\Lambda}}\tanh(\sqrt{\Lambda}t)\cos\theta\cos\phi, \frac{1}{\sqrt{\Lambda}\sin\theta}\tanh(\sqrt{\Lambda}t)\sin\phi\rangle\end{aligned}$$

Finally, taking all components to be non-zero, we have the last two Killing vector fields:

$$\begin{aligned}\xi^\mu_{(t,r,\theta,\phi),1} \to \langle &-\frac{1}{\sqrt{\Lambda}r}\sinh(\sqrt{\Lambda}t)\sin\theta\sin\phi, \cosh(\sqrt{\Lambda}t)\sin\theta\sin\phi, \frac{1}{r\cosh(\sqrt{\Lambda}t)}\cos\theta\sin\phi,\\ &\frac{1}{r\cosh(\sqrt{\Lambda}t)\sin\theta}\cos\phi\rangle\\ \xi^\mu_{(t,r,\theta,\phi),2} \to \langle &-\frac{1}{\sqrt{\Lambda}r}\sinh(\sqrt{\Lambda}t)\sin\theta\cos\phi, \cosh(\sqrt{\Lambda}t)\sin\theta\cos\phi, \frac{1}{r\cosh(\sqrt{\Lambda}t)}\cos\theta\cos\phi,\\ &-\frac{1}{r\cosh(\sqrt{\Lambda}t)\sin\theta}\sin\phi\rangle\end{aligned}$$

Summarizing, we have ten linearly independent Killing vector fields for this metric.

D Symplectic structure

Within this appendix, we'll briefly address and apply the concepts presented in [43] for the formulation of a quantum field theory of a real scalar field with a general background metric, where we shall not concern ourselves with the interaction between matter and space-time at the quantum level and instead treat the metric as non-dynamic (hence the term "background"). However, we strongly encourage the reader to review [43] in order to fully understand all the material presented below.

The information associated with the dynamical evolution of a physical system can be conveyed within the symplectic structure Ω for a particular action S given by

$$\Omega([\phi_1, \pi_1], [\phi_2, \pi_2]) = \int_{\Sigma_0} d^3x \bigg(\pi_1 \phi_2 - \pi_2 \phi_1 \bigg)$$

where Ω is a non-degenerate antisymmetric bilinear map from the solutions of the equation of motion associated with our action to the real numbers. In addition, a point in phase-space (Hamiltonian formalism) corresponds to the specification of our field solution ϕ and its conjugate momentum $\pi = \partial\mathcal{L}/\partial\dot{\phi}$ on a space-like hypersurface Σ_0 associated with our "initial value" configuration ($\mathcal{L}$ is the Lagrangian density associated with the action S which we'll give below). The fundamental Poisson brackets in classical theory can then be expressed as

$$\{\Omega([\phi_1, \pi_1], \cdot), \Omega([\phi_2, \pi_2], \cdot)\} = -\Omega([\phi_1, \pi_1], [\phi_2, \pi_2])$$

where $\Omega(y, \cdot)$ is a linear function assuming our choice of y does not vary (our input argument is only '$\cdot$'). If we arbitrarily choose $[\phi_1, \pi_1] = [0, f_1]$ and $[\phi_2, \pi_2] = [f_2, 0]$, our classical Poisson brackets reduce to

$$\bigg\{ \int d^3x f_1(x)\phi(x), \int d^3y f_2(y)\pi(y) \bigg\} = \int d^3x f_1(x) f_2(x)$$

which we can think of as the more familiar canonical relations

$$\{\phi(x), \pi(y)\} = \delta(x - y)$$

Then to construct our quantum theory of a scalar field, we extend the functions $\Omega([\phi, \pi], \cdot)$ to operators $\hat{\Omega}([\phi, \pi], \cdot)$ satisfying the commutation relations

$$[\hat{\Omega}([\phi_1, \pi_1], \cdot), \hat{\Omega}([\phi_2, \pi_2], \cdot)] = -i\hbar\Omega([\phi_1, \pi_1], [\phi_2, \pi_2])\hat{\mathrm{I}}$$

($\hat{\mathrm{I}}$ denotes the identity operator) and introduce the inner product associated with this system

$$(\psi^+, \chi^+) = -i\Omega(\bar{\psi}^+, \chi^+)$$

where $\bar{\psi}^+$ represents the complex conjugate of ψ^+ and we have decomposed our full solutions $\psi, \chi \in \mathcal{S}$ of the equation of motion for our action into

$$\psi = \psi^+ + \psi^-$$

such that our inner product with respect to these "positive frequency" solutions ψ^+, χ^+ is positive-definite. We denote the solution space spanned by these "positive frequency" parts as $\mathcal{S}^{\mathbb{C}+}$. In addition, we have expressed our inner product only in terms of solutions to our equation of motion $\psi \in \mathcal{S}$ as for each solution there corresponds a point in phase space $[\psi, \pi_\psi]$. One proceeds to "Cauchy-complete" in the norm defined by this inner product to obtain our complex Hilbert space $\mathcal{H}$ (see Chapter 3.2 and Appendix A.1 of [43]). Thus, we represent our classical observables $\Omega(\psi, \cdot)$ for each solution ψ by the operator

$$\hat{\Omega}(\psi, \cdot) = i\hat{a}(\bar{\mathrm{K}\psi}) - i\hat{a}^\dagger(\mathrm{K}\psi)$$

where $\mathrm{K} : \mathcal{S} \to \mathcal{H}$ is a map from the full solutions to our complex Hilbert space. As well, $\hat{a}(\cdot)$ and $\hat{a}^\dagger(\cdot)$ denote the annihilation and creation operators, respectively, which act on a general state Ψ in the symmetric Fock space $\mathcal{F}_s(\mathcal{H})$ in the following manner. For a general state $\Psi = \langle \psi, \psi^{a_1}, \psi^{a_1 a_2}, \dots, \psi^{a_1 \cdots a_n}, \dots \rangle$ representing our "n-particle amplitudes" where for scalar theory $\psi^{a_1 \cdots a_n} = \psi^{(a_1 \cdots a_n)}$ $\forall n$ (round parantheses denote symmetrization when dealing with abstract indices here and below) and $\xi^a \in \mathcal{H}$, $\bar{\xi}_a \in \bar{\mathcal{H}}$, we have

$$\hat{a}(\bar{\xi})\Psi = \langle \bar{\xi}_a \psi^a, \sqrt{2}\bar{\xi}_a \psi^{aa_1}, \sqrt{3}\bar{\xi}_a \psi^{aa_1,a_2}, \dots \rangle$$
$$\hat{a}^\dagger(\xi)\Psi = \langle 0, \psi\xi^{a_1}, \sqrt{2}\xi^{(a_1}\psi^{a_2)}, \sqrt{3}\xi^{(a_1}\psi^{a_2 a_3)}, \dots \rangle$$

Indices on $\bar{\xi}_a$ and ξ^a are dropped in our expressions on the left-hand side of these equations for notational convenience. In this paper, we operate under the assumption that the norms of these two expressions are finite. In addition, the inner product of two vectors $\xi, \eta \in \mathcal{H}$ is denoted by

$$(\xi, \eta) = \bar{\xi}_a \eta^a$$

In this notation, $\psi \in \otimes^n \mathcal{H}$ is denoted $\psi^{a_1 \cdots a_n}$ and $\bar{\psi} \in \otimes^n \bar{\mathcal{H}}$ as $\bar{\psi}_{a_1 \ldots a_n}$ where $\otimes^n \mathcal{H} = \mathcal{H}_1 \otimes \ldots \otimes \mathcal{H}_n$ for $\mathcal{H}_1 = \ldots = \mathcal{H}_n = \mathcal{H}$ (n-fold tensor product space). As well, $\mathcal{F}_s(\mathcal{H}) = \oplus_{n=0}^{\infty} (\otimes_s^n \mathcal{H})$ where $\otimes_s^n \mathcal{H}$ is the symmetric n-fold tensor product space and $\otimes^0 \mathcal{H}$ is defined to be the complex numbers $\mathbb{C}$ (see Appendix A of [43]).

To clarify further with regard to Fock space notation, we relate back to Dirac "bra-ket" notation:

$$|0\rangle_{\hat{a}} \equiv \langle 1, 0, 0, \ldots \rangle$$

where

$$\hat{a}(\bar{\xi})|0\rangle_{\hat{a}} = 0$$

for some general ξ^a given $|0\rangle_{\hat{a}}$ denotes the vacuum state associated with the creation and annihilation operators on our Fock space (i.e. the $\hat{a}$'s). Given our general state Ψ, the probability of finding only a single '$\hat{a}$ particle' in state $\beta \in \mathcal{H}$ is taken to be $|\bar{\beta}_a \psi^a|^2$ (i.e. ψ^a is the "one-particle amplitude", $\psi^{a_1 a_2}$ is the "two-particle amplitude", etc.). Here, the term 'particle' really refers to an excitation of the particular field associated with our $\hat{a}, \hat{a}^\dagger$ operators (quanta). Therefore, one can think of $\hat{a}(\bar{\xi})$ as an operator annihilating a quantum of state ξ^a from each of the "n-particle" states in the general state Ψ, and analogously $\hat{a}^\dagger(\xi)$ as creating a quantum in each. Our annihilation and creation operators also satisfy the commutation relation:

$$[\hat{a}(\bar{\xi}), \hat{a}^\dagger(\eta))] = \bar{\xi}_a \eta^a \hat{\mathbb{I}}$$

Note that our use of abstract index notation in this paragraph does not refer to the metric. In other words, when working with Hilbert space vectors, we always assume contraction occurs over the inner product of the respective Hilbert space as defined earlier in this appendix and not with regard to the metric.

Then for our theory of inertial centers, the action associated with the equation of motion for our Klein-Gordon extension in a particular inertial system (60) takes the form

$$S = -\frac{1}{2} \int d^4x \sqrt{|g|} \left(\nabla^a \phi \nabla_a \phi + \tilde{\mu}^2 r^2 \phi^2 \right) \tag{D.1}$$

where one can verify this by extremizing the action ($\delta S = 0$) to obtain our equation of motion. We proceed with our formulation by "slicing" our manifold M into space-like hypersurfaces each indexed by a time parameter t (Σ_t). Then, we introduce a vector field on M associated with our time evolution and defined by $t^a \nabla_a t = 1$, which we can decompose in the following manner:

$$t^a = N n^a + N^a$$

(in contrast with our previous paragraph, abstract index notation here employs the metric). n^a is the future-directed unit normal vector field to our space-like hypersurfaces Σ_t (future-directed in the sense that n^a lies in the same direction as t^a), and N^a represents the remaining tangential portion of t^a to Σ_t. In addition, we introduce coordinates t, x^1, x^2, x^3 such that $t^a \nabla_a x^i = 0$ for $i = 1, 2, 3$ which allows $t^a = (\partial/\partial t)^a$. Our action in (D.1) can then be rewritten in terms of the integral of a Lagrangian density $\mathcal{L}$ over our time parameter t and our space-like hypersurface Σ_t:

$$S = \int dt \int_{\Sigma_t} d^3x \mathcal{L}$$

with

$$\mathcal{L} = \frac{1}{2}N\sqrt{|h|}\Big((n^a\nabla_a\phi)^2 - h^{ab}\nabla_a\phi\nabla_b\phi - \tilde{\mu}^2r^2\phi^2\Big)$$

where h_{ab} is the induced Riemannian metric on Σ_t and $h = \det(h_{\beta\nu})$. Yet, since

$$n^a\nabla_a\phi = \frac{1}{N}(t^a - N^a)\nabla_a\phi = \frac{1}{N}\dot{\phi} - \frac{1}{N}N^a\nabla_a\phi$$

where $\dot{\phi} = t^a\nabla_a\phi$, we find that our conjugate momentum density on Σ_t takes the form

$$\pi = \frac{\partial\mathcal{L}}{\partial\dot{\phi}} = (n^a\nabla_a\phi)\sqrt{|h|}$$

as it does with our original Klein-Gordon action. Consequently, our symplectic structure for a free scalar field in the theory of inertial centers is given by

$$\Omega([\phi_1, \pi_1], [\phi_2, \pi_2]) = \int_{\Sigma_0} d^3x(\pi_1\phi_2 - \pi_2\phi_1)$$
$$= \int_{\Sigma_0} d^3x\sqrt{|h|}[\phi_2 n^a\nabla_a\phi_1 - \phi_1 n^a\nabla_a\phi_2]$$

where Σ_0 is the space-like hypersurface associated with our "initial value" configuration at $t = 0$.

E Divergence of a vector field

Following Chapter 3.4 of [24], the divergence of a vector field v^a is given by

$$\nabla_a v^a = \partial_a v^a + \Gamma^a_{ab}v^b$$

where we have used our knowledge from Appendix B to expand this expression. However, in component form

$$\Gamma^a_{a\nu} = \sum_\mu \Gamma^\mu_{\mu\nu} = \frac{1}{2}\sum_{\mu,\rho} g^{\mu\rho}[\partial_\mu g_{\rho\nu} + \partial_\nu g_{\rho\mu} - \partial_\rho g_{\mu\nu}]$$

Yet the first and last of these terms cancel as we are summing over both μ and ρ and $g^{ab} = g^{ba}$. This leaves us with

$$\Gamma^a_{a\nu} = \frac{1}{2}\sum_{\mu,\rho} g^{\mu\rho}\partial_\nu g_{\mu\rho}$$

but if we think in terms of the matrix form of our components, $(g_{\mu\nu})$, we have

$$\sum_{\mu,\rho} g^{\mu\rho}\partial_\nu g_{\mu\rho} = \frac{\partial_\nu g}{g}$$

where $g = \det(g_{\mu\nu})$. Therefore,

$$\Gamma^a_{a\nu} = \frac{1}{2}\frac{\partial_\nu g}{g} = \partial_\nu \ln\sqrt{|g|}$$

Plugging in above for our divergence term,

$$\nabla_a v^a = \sum_\mu \left[\partial_\mu v^\mu + v^\mu\partial_\mu \ln\sqrt{|g|}\right] = \sum_\mu \frac{1}{\sqrt{|g|}}\partial_\mu(\sqrt{|g|}v^\mu)$$

Then for a scalar field f,

$$\nabla_a\nabla^a f = \sum_\mu \frac{1}{\sqrt{|g|}}\partial_\mu(\sqrt{|g|}g^{\mu\nu}\partial_\nu f)$$

as $\nabla_a f = \partial_a f$.

F Scalar field solutions

We look for solutions (ϕ_i) to our extension of the Klein-Gordon equation:

$$(\nabla_a \nabla^a - \tilde{\mu}^2 r^2)\phi_i = 0$$

As shown in Appendix E, for a real scalar field

$$\nabla_a \nabla^a \phi_i = \sum_{\nu,\beta} \frac{1}{\sqrt{|g|}} \partial_\nu (\sqrt{|g|} g^{\nu\beta} \partial_\beta \phi_i)$$

where for our purposes $g_{\nu\beta}$ refers to the radial Rindler metric components and $\sqrt{|g|} = \sqrt{\Lambda} r^3 \cosh^2(\sqrt{\Lambda}t) \sin\theta$. Expanding (60),

$$\begin{aligned} 0 = -\tilde{\mu}^2 r^2 \phi_i - \frac{1}{\Lambda r^2 \cosh^2(\sqrt{\Lambda}t)} \partial_t (\cosh^2(\sqrt{\Lambda}t) \partial_t \phi_i) + \frac{1}{r^3} \partial_r (r^3 \partial_r \phi_i) \\ + \frac{1}{r^2 \cosh^2(\sqrt{\Lambda}t)} \left[\frac{1}{\sin\theta} \partial_\theta (\sin\theta \partial_\theta \phi_i) + \frac{1}{\sin^2\theta} \partial_\phi^2 \phi_i \right] \end{aligned} \tag{F.1}$$

We look for separable solutions of the form, $\phi_i = Z_i \cdot g(t) \cdot h(r) \cdot Y_l^m(\theta, \phi)$, where Z_i is a normalization constant and the Y_l^m are spherical harmonics satisfying

$$\frac{1}{\sin\theta} \partial_\theta (\sin\theta \partial_\theta Y_l^m) + \frac{1}{\sin^2\theta} \partial_\phi^2 Y_l^m = -l(l+1) Y_l^m \tag{F.2}$$

where

$$Y_l^m(\theta, \phi) = \sqrt{\frac{(2l+1)}{4\pi} \frac{(l-m)!}{(l+m)!}} P_l^m(\cos\theta) \cdot e^{im\phi} \tag{F.3}$$

with l as a non-negative integer, $|m| \leq l$, and m also as an integer (see Chapter 3.6 of [56] or Chapter 15.5 of [45]). P_l^m is an associated Legendre function which satisfies the differential equation

$$\left[(1-x^2)\partial_x^2 - 2x\partial_x + \left(l[l+1] - \frac{m^2}{1-x^2} \right) \right] P_l^m(x) = 0$$

and can be expressed in terms of Rodrigues' formula [46]:

$$P_l^m(x) = \frac{(-1)^m}{2^l l!} (1-x^2)^{m/2} \frac{d^{l+m}}{dx^{l+m}} (x^2-1)^l$$

with

$$P_l^{-m} = (-1)^m \frac{(l-m)!}{(l+m)!} P_l^m$$

The spherical harmonics Y_l^m as expressed above obey the orthogonality relation:

$$\int_0^\pi \sin\theta d\theta \int_0^{2\pi} d\phi Y_l^m \bar{Y}_{l'}^{m'} = \delta_{ll'} \delta_{mm'}$$

Plugging in and dividing by ϕ_i,

$$0 = -\tilde{\mu}^2 r^2 - \frac{1}{g \Lambda r^2 \cosh^2(\sqrt{\Lambda}t)} \partial_t (\cosh^2(\sqrt{\Lambda}t) \partial_t g) + \frac{1}{h r^3} \partial_r (r^3 \partial_r h) - \frac{l(l+1)}{r^2 \cosh^2(\sqrt{\Lambda}t)}$$

Multiplying through by r^2 and grouping functions of t and r:

$$\frac{1}{hr}\partial_r(r^3\partial_r h) - \tilde{\mu}^2 r^4 = \frac{1}{g\Lambda\cosh^2(\sqrt{\Lambda}t)}\partial_t(\cosh^2(\sqrt{\Lambda}t)\partial_t g) + \frac{l(l+1)}{\cosh^2(\sqrt{\Lambda}t)} = -(4\alpha^2+1)$$

where α is a constant. Thus, we have two differential equations:

$$0 = r^2\partial_r^2 h + 3r\partial_r h + \left[(4\alpha^2+1) - \tilde{\mu}^2 r^4\right]h \tag{F.4}$$

$$0 = \frac{1}{\Lambda\cosh^2(\sqrt{\Lambda}t)}\partial_t(\cosh^2(\sqrt{\Lambda}t)\partial_t g) + \left[\frac{l(l+1)}{\cosh^2(\sqrt{\Lambda}t)} + (4\alpha^2+1)\right]g \tag{F.5}$$

Focusing on our radial equation first, we set $\rho = \sqrt{\tilde{\mu}}r$:

$$0 = \rho^2\partial_\rho^2 h + 3\rho\partial_\rho h + \left[(4\alpha^2+1) - \rho^4\right]h \tag{F.6}$$

Letting $h(\rho) = z(\rho)/\rho$

$$0 = \rho^2\partial_\rho^2 z + \rho\partial_\rho z + \left[4\alpha^2 - \rho^4\right]z$$

and setting $y = \rho^2/2$, we find

$$0 = y^2\partial_y^2 z + y\partial_y z + [\alpha^2 - y^2]z$$

But this is just the modified Bessel equation of pure imaginary order (see Chapter 3 of [47]). Choosing the physically realistic solution (we expect h to decay for large r since in our classical analysis massive objects are "confined" to motion about their inertial centers), our full expression takes the form

$$h_\alpha(\rho) = \frac{K_{i\alpha}(\frac{\rho^2}{2})}{\rho} \tag{F.7}$$

where $K_{i\alpha}$ is the Macdonald function (modified Bessel function) of imaginary order α given in integral form (for $y > 0$):

$$K_{i\alpha}(y) = \int_0^\infty d\eta\cos(\alpha\eta)e^{-y\cosh\eta}$$

and α is restricted to the range: $0 \leq \alpha < \infty$ (see Chapter 4.15 of [48] and [42] for its application to quantization in the classic Rindler case with the Klein-Gordon equation).

The Macdonald function of imaginary order obeys an orthogonality relation which will be useful to us for determining part of our normalization constant. From [49],

$$\int_0^\infty dy\frac{K_{i\alpha}(y)K_{i\alpha'}(y)}{y} = \frac{\pi^2}{2\alpha\sinh(\pi\alpha)}\delta(\alpha-\alpha')$$

Later we'll need:

$$\begin{aligned}\int_0^\infty d\rho\frac{K_{i\alpha}(\frac{\rho^2}{2})K_{i\alpha'}(\frac{\rho^2}{2})}{\rho} &= \int_0^\infty d\eta\int_0^\infty d\eta'\cos(\alpha\eta)\cos(\alpha'\eta')\int_0^\infty\frac{d\rho}{\rho}e^{-(\rho^2/2)(\cosh\eta+\cosh\eta')} \\ &= \int_0^\infty d\eta\int_0^\infty d\eta'\cos(\alpha\eta)\cos(\alpha'\eta')\int_0^\infty\frac{dy}{2y}e^{-y(\cosh\eta+\cosh\eta')} \\ &= \frac{1}{2}\int_0^\infty dy\frac{K_{i\alpha}(y)K_{i\alpha'}(y)}{y} = \frac{\pi^2}{4\alpha\sinh(\pi\alpha)}\delta(\alpha-\alpha')\end{aligned}$$

Examining our second differential equation, we let $\eta = \tanh(\sqrt{\Lambda}t)$:

$$0 = (1-\eta^2)^2\partial_\eta^2 g + [l(l+1)(1-\eta^2) + (4\alpha^2+1)]g \tag{F.8}$$

where $\eta^2 < 1$. Before proceeding any further, we must make one remark that will be crucial for evaluating our inner product. Taking the complex conjugate of (F.8)

$$0 = (1-\eta^2)^2\partial_\eta^2 \bar{g} + [l(l+1)(1-\eta^2) + (4\alpha^2+1)]\bar{g}$$

Multiplying (F.8) by $\bar{g}$ and subtracting by g times the complex conjugate of (F.8), we find

$$\bar{g}\partial_\eta^2 g - g\partial_\eta^2 \bar{g} = 0$$

or

$$\bar{g}\partial_\eta g - g\partial_\eta \bar{g} = \text{constant} \tag{F.9}$$

Returning to (F.8), we divide through by $1-\eta^2$ and make the substitution $g(\eta) = \sqrt{1-\eta^2}\cdot p(\eta)$:

$$0 = (1-\eta^2)\sqrt{1-\eta^2}\left[\partial_\eta^2 p - \frac{2\eta}{1-\eta^2}\partial_\eta p - \frac{p}{(1-\eta^2)^2}\right] + \left[l(l+1) + \frac{4\alpha^2}{1-\eta^2} + \frac{1}{1-\eta^2}\right]\sqrt{1-\eta^2}\cdot p$$

which reduces to

$$0 = (1-\eta^2)\partial_\eta^2 p - 2\eta\partial_\eta p + \left[l(l+1) + \frac{4\alpha^2}{1-\eta^2}\right]p$$

But the solution to this differential equation is the Legendre function of the first kind [46] (since our domain is restricted to $\eta^2 < 1$) which can be expressed in the following manner:

$$P_l^{\pm 2i\alpha}(\eta) = \frac{1}{\Gamma(1\mp 2i\alpha)}\left[\frac{1+\eta}{1-\eta}\right]^{\pm i\alpha} {}_2F_1(-l, l+1; 1\mp 2i\alpha, \frac{1-\eta}{2}) \tag{F.10}$$

where ${}_2F_1$ is the hypergeometric function which for our parameters can take the form

$${}_2F_1(-l, l+1; 1\mp 2i\alpha, \frac{1-\eta}{2}) = \frac{\Gamma(1\mp 2i\alpha)}{\Gamma(-l)\Gamma(l+1)}\sum_{k=0}^{\infty}\frac{\Gamma(k-l)\Gamma(k+l+1)}{k!\cdot\Gamma(k+1\mp 2i\alpha)}\left(\frac{1-\eta}{2}\right)^k \tag{F.11}$$

and $\Gamma(z)$ is the gamma function which can be written in integral form for $\Re(z) > 0$ where z is a complex variable as

$$\Gamma(z) = \int_0^\infty t^{z-1}e^{-t}dt$$

Note that this Legendre function, P_ν^μ, is in fact a generalization of the Legendre function used earlier for our angular dependence where the parameters μ, ν here are allowed to be complex numbers instead of solely real integers. Our full expression for g is then

$$g(\eta) = \sqrt{1-\eta^2}\cdot P_l^{-2i\alpha}(\eta) \tag{F.12}$$

where as we'll see below, our choice of $-2i\alpha$ is necessary in order to ensure that our inner product is positive-definite with respect to our solutions for ϕ_i so that we may properly construct our field operator (i.e. we take the "positive frequency" solutions; see Chapter 3.2 of [43]).

To find our normalization constant, we'll need to evaluate (F.9). For the rest of our analysis in this appendix, we use Chapter 8 of [46] as a reference for our general expressions. Beginning with $\partial_\eta g$:

$$\partial_\eta g = -\frac{\eta}{\sqrt{1-\eta^2}}P_l^{-2i\alpha} + \sqrt{1-\eta^2}\partial_\eta P_l^{-2i\alpha}$$

But

$$\partial_\eta P_\nu^\mu = -\frac{\nu\eta}{1-\eta^2}P_\nu^\mu + \frac{\mu+\nu}{1-\eta^2}P_{\nu-1}^\mu$$

Plugging in above

$$\begin{aligned}\partial_\eta g &= -\frac{\eta}{\sqrt{1-\eta^2}}P_l^{-2i\alpha} + \sqrt{1-\eta^2}\left(-\frac{l\eta}{1-\eta^2}P_l^{-2i\alpha} + \frac{l-2i\alpha}{1-\eta^2}P_{l-1}^{-2i\alpha}\right)\\ &= -\frac{\eta(1+l)}{\sqrt{1-\eta^2}}P_l^{-2i\alpha} + \frac{l-2i\alpha}{\sqrt{1-\eta^2}}P_{l-1}^{-2i\alpha}\end{aligned}$$

Then

$$\begin{aligned}\bar{g}\partial_\eta g - g\partial_\eta\bar{g} = \sqrt{1-\eta^2}\Bigg[&\bar{P}_l^{-2i\alpha}\left(-\frac{\eta(1+l)}{\sqrt{1-\eta^2}}P_l^{-2i\alpha} + \frac{l-2i\alpha}{\sqrt{1-\eta^2}}P_{l-1}^{-2i\alpha}\right)\\ &-P_l^{-2i\alpha}\left(-\frac{\eta(1+l)}{\sqrt{1-\eta^2}}\bar{P}_l^{-2i\alpha} + \frac{l+2i\alpha}{\sqrt{1-\eta^2}}\bar{P}_{l-1}^{-2i\alpha}\right)\Bigg]\\ &= (l-2i\alpha)\bar{P}_l^{-2i\alpha}P_{l-1}^{-2i\alpha} - (l+2i\alpha)P_l^{-2i\alpha}\bar{P}_{l-1}^{-2i\alpha}\end{aligned}$$

However, as one can tell from (F.10), $\bar{P}_l^{-2i\alpha} = P_l^{2i\alpha}$. Therefore,

$$\bar{g}\partial_\eta g - g\partial_\eta\bar{g} = (l-2i\alpha)P_l^{2i\alpha}P_{l-1}^{-2i\alpha} - (l+2i\alpha)P_l^{-2i\alpha}P_{l-1}^{2i\alpha} = \text{constant}$$

Yet since this expression must be constant, we can evaluate for any particular value of η. Because we have expressions for the Legendre functions at $\eta = 0$, we'll make this convenient choice where

$$P_\nu^\mu(0) = 2^\mu\pi^{-1/2}\cos\left[\frac{\pi}{2}(\nu+\mu)\right]\frac{\Gamma(\frac{1}{2}+\frac{1}{2}\nu+\frac{1}{2}\mu)}{\Gamma(1+\frac{1}{2}\nu-\frac{1}{2}\mu)}$$

For $\eta = 0$:

$$\begin{aligned}P_{l-1}^{-2i\alpha}(0)\cdot P_l^{2i\alpha}(0) &= 2^{-2i\alpha}\pi^{-1/2}\cos\left[\frac{\pi}{2}(l-1-2i\alpha)\right]\frac{\Gamma(\frac{1}{2}+\frac{l-1}{2}-i\alpha)}{\Gamma(1+\frac{l-1}{2}+i\alpha)}\\ &\cdot 2^{2i\alpha}\pi^{-1/2}\cos\left[\frac{\pi}{2}(l+2i\alpha)\right]\frac{\Gamma(\frac{1}{2}+\frac{l}{2}+i\alpha)}{\Gamma(1+\frac{l}{2}-i\alpha)}\\ &= \frac{1}{\pi}\cos\left[\frac{\pi}{2}(l-1-2i\alpha)\right]\cos\left[\frac{\pi}{2}(l+2i\alpha)\right]\frac{\Gamma(\frac{l}{2}-i\alpha)}{\Gamma(1+\frac{l}{2}-i\alpha)}\end{aligned}$$

But from properties of the gamma function (see Chapter 6 of [46]),

$$\Gamma(1+z) = z\Gamma(z)$$

Using this property above,

$$P_{l-1}^{-2i\alpha}(0)\cdot P_l^{2i\alpha}(0) = \frac{1}{\pi(\frac{l}{2}-i\alpha)}\cos\left[\frac{\pi}{2}(l-1-2i\alpha)\right]\cos\left[\frac{\pi}{2}(l+2i\alpha)\right]$$

And with the expressions

$$\begin{aligned}\cos\left[\frac{\pi}{2}(l-1-2i\alpha)\right] &= \frac{1}{2}\left[e^{i(\pi/2)(l-1-2i\alpha)} + e^{-i(\pi/2)(l-1-2i\alpha)}\right]\\ \cos\left[\frac{\pi}{2}(l+2i\alpha)\right] &= \frac{1}{2}\left[e^{i(\pi/2)(l+2i\alpha)} + e^{-i(\pi/2)(l+2i\alpha)}\right]\end{aligned}$$

we have

$$\cos\left[\frac{\pi}{2}(l-1-2i\alpha)\right]\cos\left[\frac{\pi}{2}(l+2i\alpha)\right]=\frac{1}{4}\left[e^{i\pi(l-1/2)}+e^{-i\pi(l-1/2)}+e^{i\pi(2i\alpha+1/2)}+e^{-i\pi(2i\alpha+1/2)}\right]$$

Thus,

$$(l-2i\alpha)P_{l-1}^{-2i\alpha}P_l^{2i\alpha}|_{\eta=0}=\frac{1}{2\pi}\left[e^{i\pi(l-1/2)}+e^{-i\pi(l-1/2)}+e^{i\pi(2i\alpha+1/2)}+e^{-i\pi(2i\alpha+1/2)}\right]$$

Applying similar logic to the second term in our expression above, we find

$$(l+2i\alpha)P_l^{-2i\alpha}P_{l-1}^{2i\alpha}|_{\eta=0}=\frac{1}{2\pi}\left[e^{i\pi(l-1/2)}+e^{-i\pi(l-1/2)}+e^{i\pi(2i\alpha-1/2)}+e^{-i\pi(2i\alpha-1/2)}\right]$$

Then

$$\begin{aligned}\bar{g}\partial_\eta g-g\partial_\eta\bar{g}&=\frac{1}{2\pi}\left[e^{i\pi/2-2\pi\alpha}+e^{-i\pi/2+2\pi\alpha}-e^{i\pi/2+2\pi\alpha}-e^{-i\pi/2-2\pi\alpha}\right]\\&=\frac{1}{2\pi}\left[e^{i\pi/2}\left(e^{-2\pi\alpha}-e^{2\pi\alpha}\right)+e^{-i\pi/2}\left(e^{2\pi\alpha}-e^{-2\pi\alpha}\right)\right]\\&=-\frac{(e^{2\pi\alpha}-e^{-2\pi\alpha})(e^{i\pi/2}-e^{-i\pi/2})}{2\pi}\end{aligned}$$

But

$$e^{i\pi/2}-e^{-i\pi/2}=2i\sin(\pi/2)=2i$$

and

$$e^{2\pi\alpha}-e^{-2\pi\alpha}=2\sinh(2\pi\alpha)=4\sinh(\pi\alpha)\cosh(\pi\alpha)$$

Therefore, plugging in above

$$\bar{g}\partial_\eta g-g\partial_\eta\bar{g}=-\frac{4i}{\pi}\sinh(\pi\alpha)\cosh(\pi\alpha)$$

Addressing our inner product where our solutions are of the form $\phi_i = Z_i \cdot g \cdot h \cdot Y_l^m$

$$\begin{aligned}
(\phi_1, \phi_2) = -\frac{i}{\sqrt{\Lambda}} Z_1 Z_2 \cosh^2(\sqrt{\Lambda}t)[g_2 \partial_t \bar{g}_1 - \bar{g}_1 \partial_t g_2]|_{t=0} \int_0^{\pi} \sin\theta d\theta \int_0^{2\pi} d\phi \bar{Y}_{l_1}^{m_1} Y_{l_2}^{m_2} \\
\cdot \Big[\int_0^{\infty} r\bar{h}_{\alpha_1} h_{\alpha_2} dr - \int_{-\infty}^{0} r\bar{h}_{\alpha_1} h_{\alpha_2} dr \Big] \\
= iZ_1 Z_2 \delta_{l_1 l_2} \delta_{m_1 m_2} [\bar{g}_1 \partial_\eta g_2 - g_2 \partial_\eta \bar{g}_1]|_{\eta=0} \cdot \Big[\int_0^{\infty} dr \frac{K_{i\alpha_1}(\frac{\tilde{\mu}r^2}{2}) K_{i\alpha_2}(\frac{\tilde{\mu}r^2}{2})}{\tilde{\mu}r} \\
- \int_{-\infty}^{0} dr \frac{K_{i\alpha_1}(\frac{\tilde{\mu}r^2}{2}) K_{i\alpha_2}(\frac{\tilde{\mu}r^2}{2})}{\tilde{\mu}r} \Big] \\
= iZ_1 Z_2 \delta_{l_1 l_2} \delta_{m_1 m_2} [\bar{g}_1 \partial_\eta g_2 - g_2 \partial_\eta \bar{g}_1]|_{\eta=0} \cdot \frac{2}{\tilde{\mu}} \int_0^{\infty} d\rho \frac{K_{i\alpha_1}(\frac{\rho^2}{2}) K_{i\alpha_2}(\frac{\rho^2}{2})}{\rho} \\
= iZ_1 Z_2 \delta_{l_1 l_2} \delta_{m_1 m_2} \delta(\alpha_1 - \alpha_2) \cdot \frac{\pi^2}{2\tilde{\mu}\alpha_1 \sinh(\pi\alpha_1)} \cdot [\bar{g}_1 \partial_\eta g_2 - g_2 \partial_\eta \bar{g}_1]|_{\eta=0} \\
= iZ_1^2 \delta_{l_1 l_2} \delta_{m_1 m_2} \delta(\alpha_1 - \alpha_2) \cdot \frac{\pi^2}{2\tilde{\mu}\alpha_1 \sinh(\pi\alpha_1)} \cdot [\bar{g}_1 \partial_\eta g_1 - g_1 \partial_\eta \bar{g}_1]|_{\eta=0} \\
= iZ_1^2 \delta_{l_1 l_2} \delta_{m_1 m_2} \delta(\alpha_1 - \alpha_2) \cdot \frac{\pi^2}{2\tilde{\mu}\alpha_1 \sinh(\pi\alpha_1)} \Big[-\frac{4i \sinh(\pi\alpha_1) \cosh(\pi\alpha_1)}{\pi} \Big] \\
= Z_1^2 \delta_{l_1 l_2} \delta_{m_1 m_2} \delta(\alpha_1 - \alpha_2) \cdot \frac{2\pi \cosh(\pi\alpha_1)}{\tilde{\mu}\alpha_1}
\end{aligned}$$

where we keep in mind the fact that

$$\frac{\partial}{\partial t} = \frac{\partial \eta}{\partial t} \frac{\partial}{\partial \eta} = \frac{\sqrt{\Lambda}}{\cosh^2(\sqrt{\Lambda}t)} \cdot \frac{\partial}{\partial \eta}$$

Thus, our normalization constant is found to be

$$Z_\alpha = \sqrt{\frac{\tilde{\mu}\alpha}{2\pi \cosh(\pi\alpha)}} \tag{F.13}$$
